# Characterization of the transposable element landscape shaping the *Ectocarpus* genome

**DOI:** 10.1186/s13059-025-03742-z

**Published:** 2025-09-29

**Authors:** Erica Dinatale, Rory J. Craig, Claudia Martinho, Hajk-Georg Drost, Susana M. Coelho

**Affiliations:** 1 Department of Algal Development and Evolution, Max Planck Institute for Biology, Tübingen, Germany; 2 Present Address: School of Life Sciences, Division of Plant Sciences, University of Dundee, James Hutton Institute, Invergowrie, Dundee, Scotland, UK DD25DA; 3 Computational Biology Group, Max Planck Institute for Biology, Tübingen, Germany; 4 Digital Biology Group, Division of Computational Biology, School of Life Sciences, University of Dundee, Dundee, UK; 5 School of BioSciences, University of Melbourne, Parkville, Australia VIC 3010

## Abstract

**Background:**

Comprising up to 90% of eukaryotic genomes, transposable elements (TEs) are mobile genetic units that play fundamental roles in evolution. Brown algae, one of the most complex multicellular eukaryotic groups that evolved independently from plants, fungi, and animals, are particularly underexplored in their transposon biology, especially when studied in a developmental context.

**Results:**

Here, we explore the TE landscape of the model brown alga *Ectocarpus*, using a high-quality genome assembly complemented by extensive manual curation. TEs account for 28% of the genome, with a predominance of evolutionarily young elements. DNA transposons represent the most abundant and diverse TE subclass. Notably, TEs are significantly enriched along the sex chromosomes, a pattern potentially driven by local transposition events from the non-recombining sex-determining region into the pseudoautosomal regions. The genome harbors a high density of intronic TEs, which show minimal impact on host gene expression; however, intronic TEs tend to be shorter and more degraded than intergenic copies, suggesting selective pressures on their retention in the genome. Intact and potentially active TEs are preferentially associated with small RNAs and the histone modification H3K79me2, with over 70% of H3K79me2-marked intact TEs also enriched in small RNAs. This stable association indicates tight and sustained silencing of intact TEs throughout the life cycle of Ectocarpus.

**Conclusions:**

Our study highlights the genetic diversity of the *Ectocarpus* mobilome and presents a complex, multilayered landscape of TE regulation mechanisms which involves small RNAs and chromatin modifications in the absence of an epigenetic silencing machinery that would be comparable to animals or plants.

**Supplementary Information:**

The online version contains supplementary material available at 10.1186/s13059-025-03742-z.

## Background

Brown algae are one of the most complex multicellular lineages on Earth, only distantly related to animals, land plants, and fungi, from which they diverged over 1 billion years ago [1, 2]. They have colonized various marine habitats and evolved diverse morphologies, developmental patterns, life cycles, and sex determination systems [3, 4]. *Ectocarpus* is a filamentous brown alga that has emerged as a model organism representing this group of eukaryotes [5, 6]. *Ectocarpus* undergoes a haploid-diploid life cycle, alternating between haploid gametophyte, in which sex is determined by the presence of a male (V) or female (U) sex chromosome, and diploid sporophyte generations. Both generations are multicellular and relatively simple in terms of development [7]. Although significant progress has been made in understanding development, sex determination, and gene regulation in the *Ectocarpus* genome (reviewed in [7, 8]), relatively little is known about mobile genetic elements in this species. This lack of knowledge extends across brown algae, hindering our understanding of how mobile elements have influenced the evolution of this major multicellular lineage.

Transposable elements (TEs) are mobile genetic units that display substantial mechanistic diversity and are near-ubiquitous across the tree of life [9]. TEs can copy or excise their sequence and insert at new genomic locations, with this replicative nature frequently leading to their accumulation in genomes. Both the proportion and diversity of TEs in a host genome can vary dramatically, even between closely related species [10, 11]. TE abundance also differs between and within chromosomes, and the genomic distribution of different TEs is influenced by multiple forces, including the strength of selection against TE insertions, the local recombination landscape, and TE insertion biases [12, 13]. In brown algae, estimated repeat contents range from ~ 20% in *Ectocarpus* to 70% (e.g., the Fucales and Dictyotales), and variation in the abundance of repeats largely explains differences in genome size [2]. TEs are enriched on the U and V sex chromosomes, especially in the sex-determining region (SDR) [4, 14]. Yet, in contrast to many animal, plant and fungal systems, most TE families in the *Ectocarpus* genome, and consequently across brown algae, remain unclassified and largely uncharacterized.

TEs have long been considered selfish genetic elements and threats to genome stability [15, 16], or as benign “junk” DNA that accumulates in genomes as degraded and inactive copies [9, 17]. However, in a return to the foundational hypotheses of transposon biology [18], it is now clear that TEs play many fundamental roles in evolution. For example, TE insertions can induce phenotypic change and adaptation, and TE co-option acts as a major driving force of gene regulatory element evolution [10, 19]. Nevertheless, TEs are believed to be inherently mutagenic and eukaryotes have evolved elaborate mechanisms to prevent or control the proliferation of TEs.

Broadly, TE silencing mechanisms include transcriptional and post-transcriptional inhibition of the TE sequences and genes required for their replication and mobilization. These mechanisms frequently involve repressive epigenetic modifications and small RNA (sRNA) molecules with high sequence complementarity to a TE target region, which can function either independently or in concert. In flowering plants, sRNAs are involved in the establishment of repressive DNA and chromatin modifications at TE loci, and also direct small interfering RNA-mediated cleavage of TE mRNA [20, 21]. In animals, TE transcripts can also be the target of small interfering RNAs (siRNAs), while PIWI-interacting RNAs (piRNAs) repress both TE transcripts and genomic loci [22]. In ciliates, sRNAs guide the removal of TEs from the macronucleus [23]. Repressive modifications include DNA methylation (especially 5’ methylcystosine; 5mC) and histone post-translational modifications that reduce chromatin accessibility. In particular, in plants, animals and some ciliate species, trimethylation of histone H3 lysine 27 (H3K27me3) and mono-, di- and trimethylation of histone H3 lysine 9 (H3K9me1/2/3) are associated with transcriptional suppression and TE silencing [24–26].

How TEs are silenced in brown algae is largely unknown. *Ectocarpus* displays negligible levels of 5mC, both genome-wide and on TEs [27]. Furthermore, classical repressive histone marks, including H3K27me3 and H3K9me1/2/3, are absent in the species. The histone marks H3K79me2 and H4K20me3 have been associated with decreased transcript abundance [28–30] and repeats [31]. In particular, 88.6% of H3K79me2 regions of > 5 kbp included a ≥ 400-bp repeated element, suggesting a potential role for this mark in TE control [31]. Furthermore, *Ectocarpus* sRNAs show an association with TEs [27]. sRNAs exhibit a predominance for 21-nt species, including microRNA families [32].

Here, we combine manual TE curation with a recent chromosome-level genome assembly [33] to comprehensively characterize the *Ectocarpus* mobilome and investigate TE regulation across its life cycle. We show that the majority of TEs in the *Ectocarpus* genome are evolutionarily young, with diverse DNA transposons representing the most abundant subclass and contributing significantly to repeat enrichment on the sex chromosome. Using integrated ChIP-seq, sRNA-seq, and RNA-seq datasets, we further demonstrate that intact and potentially active TEs are preferentially associated with putative silencing mechanisms, suggesting a tightly regulated control of TE activity throughout development.

## Results

### The Ectocarpus genome features a high diversity of recently active TEs

To characterize the genomic landscape and diversity of TEs in *Ectocarpus* sp. 7 (hereafter *Ectocarpus*), we used the latest high-quality, near-gapless genome assembly, which is based on a haploid male individual (strain Ec32 [33],) and spans 200.2 Mb at telomere-to-telomere level for many chromosomes [33]. This highly contiguous reference genome provides the opportunity to characterize TE dynamics at unprecedented resolution in a brown alga.

An existing *Ectocarpus* TE library features 897 consensus sequences [27] and was generated using the automated repeat annotation tool REPET [34]. Following a stringent post-processing and quality control procedure for these 897 sequences (see Methods), we detected high levels of redundancy, mis-classification, and lack of classification (i.e., unknown repeats). Repeat classification is particularly challenging in taxonomically diverse species, since the process largely relies on homology to TEs and TE proteins from species with curated TE libraries [35]. To overcome these limitations, we produced a de novo TE library complemented by a manual curation procedure, primarily targeting the most abundant elements (i.e., TE families contributing at least ~ 100 kb to the genome) (Methods). This annotation and curation effort yielded 165 verified consensus sequences, each of which served as a representative for a single autonomous or nonautonomous TE family (or subfamily, if relevant) (Supplementary Material 1 and 2). For the remaining low abundance TE models from the REPET library, we performed sequence clustering analysis to remove redundancy and performed additional re-classification via homology comparisons to the manually curated models (see Methods). The final library was produced by combining the manual TE models with the non-redundant REPET models, yielding 574 consensus sequences, corresponding to 347 families (Table 1, see Supplementary Material 3 for detailed annotation notes for TE families).

**Table 1:** Summary statistics of TE superfamilies in the *Ectocarpus* genome

Category	Families	Copies	Intact copies	Genome coverage (%)
LINE/*CRE*	2	4821	30	0.42%
LINE/*RTE*	3	5010	188	0.97%
LINE/*RTE-X*	7	16,418	4100	3.41%
LINE/Unknown	3	641	16	0.14%
LTR/*Copia*	35	5023	561	3.00%
LTR/*Gypsy*	32	4221	196	2.78%
LTR/Unknown	26	1144	198	0.47%
DIRS/*Ngaro*	15	3893	689	2.45%
DIRS/*PAT-like*	5	842	63	0.58%
PLE/*Chlamys*	3	4782	27	0.43%
DNA/Unknown	51	9109	1308	1.70%
DNA/*Dada*	1	15	1	0.01%
DNA/*Harbinger*	15	2251	916	0.93%
DNA/*KDZ*	8	6403	148	1.80%
DNA/*Mariner-Tc1*	17	11,912	3007	1.43%
DNA/*MuDR*	1	56	42	0.13%
DNA/*PiggyBac*	4	1588	289	0.48%
DNA/*Sola-1*	1	1989	2	0.17%
DNA/*Sola-2*	1	48	14	0.04%
DNA/*hAT*	4	128	13	0.05%
DNA/*EnSpm*	5	6366	161	1.11%
Helitron	13	4688	522	1.10%
Unknown	94	22,127	4037	3.93%
Tandem repeats	/	/	/	3.35%
Total	393	113,475	16,528	30.91%

**Fig. 1:**
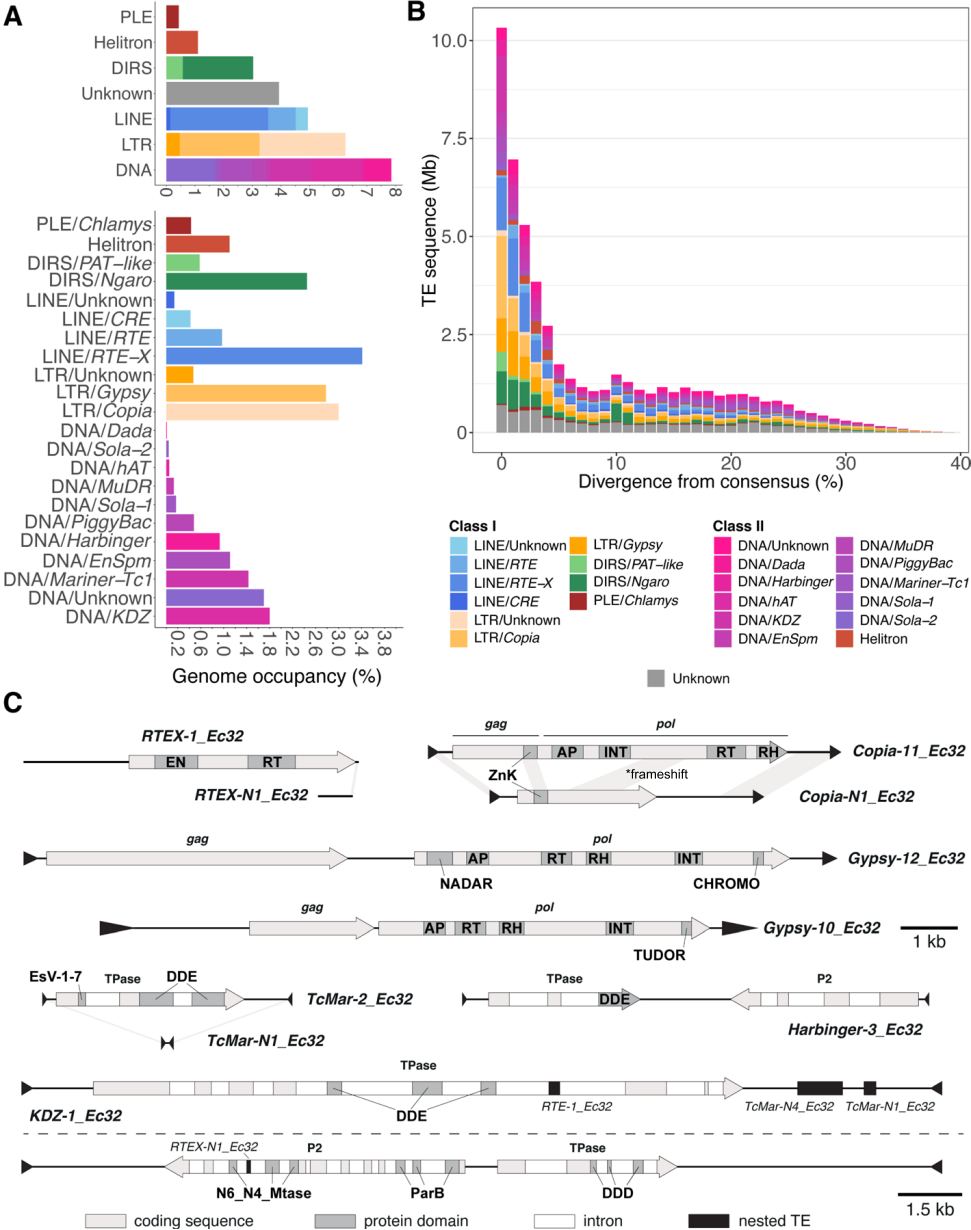
Genomic occupancy and landscape of 113,475 annotated TEs (574 consensus sequences) in *Ectocarpus*. **A** Percentage of the genome spanned by different subclasses and superfamilies of TEs. DNA refers to DD(E/D) transposons. **B** TE landscape showing cumulative genomic TE content relative to divergence from TE family consensus sequence (a proxy for TE age). **C** Schematics of *Ectocarpus* TEs showing terminal motifs, genes, and protein domains. Models are to scale except for the terminal inverted repeats of DNA transposons which are enlarged for visualization. Domain abbreviations are: EN = endonuclease, RT = reverse transcriptase, INT = integrase, RH = RNaseH, ZnK = Gag zinc knuckle, AP = aspartic protease, DDE = DD(E/D)-type integrase

The *Ectocarpus* genome harbors considerable TE diversity. Most of the 347 TE families correspond to the major subclasses of eukaryotic TEs, which can broadly be grouped as either class I (moving via an RNA intermediate) or class II (a DNA intermediate) [36]. The *Ectocarpus* genome features all class I retrotransposon subclasses: LTR retrotransposons, long interspersed nuclear elements (LINEs), tyrosine recombinase retrotransposons (DIRS), and *Penelope*-like elements (PLEs) [9, 37]. For Class II, our curation revealed substantial diversity in DD(E/D) DNA transposons (i.e., canonical *cut*-and-*paste* DNA transposons, hereafter simply DNA transposons), in addition to Helitron elements that move via a *copy*-and-*paste* rolling circle mechanism [38]. We identified individual TE copies in the *Ectocarpus* genome by passing our library to RepeatMasker, and also identified satellite and microsatellite arrays using Tandem Repeats Finder. Overall, repeats constitute 31% of the *Ectocarpus* genome, the vast majority of which are TEs (28% of the genome, i.e., 55.2 Mb). DNA transposons account for 7.9%, LTR retrotransposons 6.3%, and LINEs 4.9% (Fig. 1A, Table 1). The TE landscape is largely comprised of copies with high sequence similarity within a given family, with 53% of the total genomic TE content attributable to TE copies exhibiting < 5% divergence from their family consensus sequence (Fig. 1B). This broad lack of degradation suggests that most TE families in the *Ectocarpus* genome may have been recently active, and that many TE copies may have retained the competency to transpose.

At the level of individual families, the most abundant TEs are LINEs and non-autonomous elements that putatively rely on autonomous LINEs for their transposition (Supplementary Material 4). Specifically, multiple families from the *RTE-X*, *RTE*, and *CRE* clades are each present in several thousand individual copies (Supplementary Material 4). The most abundant single family, present in more than 6000 copies and totaling 2.48 Mb of the genome, is a ~ 600 bp non-autonomous element (*RTEX-N1_Ec32*) that exhibits sequence conservation at its 3’ end to autonomous *RTE-X* elements (*RTEX-1* and *RTEX-2_Ec32*, Fig. 1C). It is possible that *RTEX-N1_Ec32* is a SINE (short interspersed nuclear element); however, we were unable to determine the origin of its 5’ region. For simplicity, we group this family and all other similar families with LINEs, since some classification systems require SINEs to have RNA polymerase III promoters [39].

Although less abundant as individual families, LTR retrotransposons exhibit a greater family-level diversity than LINEs and collectively constitute more sequence in the *Ectocarpus* genome (Fig. 1A, B). All *Ectocarpus* LTR retrotransposons belong to the *Metaviridae* (i.e., *Ty3/Gypsy*, 32 families) and *Pseudoviridae* (i.e., *Ty1*/*Copia*, 35 families) groups. As previously reported in *Ectocarpus* [27] and some plant genomes [40], we found several *Copia* families that carry a single ORF with at least partial homology to the *gag* ORF of fully autonomous elements (e.g., *Copia-N1_Ec32* and *Copia-11_Ec32*, Fig. 1C). *Gypsy* families from the *Ectocarpus* genome display considerable structural diversity, and phylogenetic analysis revealed four distinct lineages distributed among the diversity of eukaryotic *Gypsy* elements (Suplementary Material 5: Fig. S1). Notably, families from all four lineages encode polyproteins featuring chromatin reader domains that are associated with chromatin recognition, remodeling, and targeted insertion. Families from three of the lineages feature putative C-terminal chromodomains fused to the integrase domain of Pol (e.g., *Gypsy-12_Ec32*, Fig. 1C). One of these lineages corresponds to *ECR-1*, which we previously described as an *Ectocarpus* centromere-specific element [33]. Interestingly, the *Ectocarpus* chromodomain-containing *Gypsy* lineages are not phylogenetically related to chromoviruses (the major group of chromodomain-containing *Gypsy* elements [41]) nor the Chronos chromodomain-containing *Gypsy* elements of oomycetes [42] (Supplementary Material 7: Fig. S1). The final lineage corresponds to an element carrying a C-terminal Tudor domain (*Gypsy-10_Ec32*, Fig. 1C), which together with chromodomains form part of a larger family of histone readers [43]. To our knowledge, this is the first reported TE that carries a Tudor domain. We recovered a Tudor domain-containing homolog of *Gypsy-10_Ec32* in the yellow-green alga *Tribonema minus*, suggesting acquisition of this domain prior to the origin of brown algae more than 450 mya [44]. Collectively, our results support multiple independent acquisitions of chromatin reader domains by *Gypsy* elements in stramenopiles, potentially indicating unique interactions between retroelements and the host chromatin-landscape in this evolutionary lineage.

Furthermore, the families from one of the four *Gypsy* lineages all feature a highly conserved YbiA-family NADAR domain at the N-terminal region of Pol (e.g. *Gypsy-12_Ec32*, Fig. 1C; Supplementary Material 7: Fig. S2). YbiA-family NADAR (NAD and ADP-ribose) domain proteins are present in prokaryotes, eukaryotes, and viruses and play diverse roles in enzymatic pathways involving NAD (nicotinamide adenine dinucleotide) and ADP-ribose, including processing of nucleic acids and proteins [45]. Excluding *Ectocarpus*, blastp queries of the *Gypsy* NADAR domains almost exclusively retrieved bacterial hits, suggesting possible acquisition of the domain by horizontal transfer (Supplementary Material 8: Table S1). Querying the Repbase database of TE proteins recovered divergent hits to the ORF1 proteins from several families of *Tx1* LINEs from the Pacific Oyster *Crassostrea gigas*, which also appear to constitute YbiA-family NADAR domains (Supplementary Material 7: Fig. S2). Although their role in retrotransposition is unclear, the presence of YbiA-family NADAR domains in RNA viruses has been hypothesized to function in RNA processing [45] and it may be the case that the NADAR domains are involved in processing the RNA intermediates of transposition in these *Ectocarpus* and *C. gigas* elements.

The remaining retrotransposons belong to the *Ngaro* and *PAT-like* groups of DIRS elements, and the *Chlamys* group of PLEs. *Ngaro* elements are particularly diverse (15 families) and abundant (2.45% of the genome) (Fig. 1A). PLEs have not previously been reported in TSAR (telonemids-stramenopiles-alveolates-rhizaria) [46], and their discovery in *Ectocarpus* extends their known range to a new eukaryotic supergroup.

DNA transposons are both the most abundant and most diverse subclass of TEs in *Ectocarpus*, with at least 10 superfamilies present (Fig. 1A, B). *Harbinger* (15 families) and *Mariner*-*Tc1* (17 families) are the most diverse DNA transposons at the family level, and *Mariner-Tc1* DNA transposons collectively contribute almost 12,000 genomic copies (Table 1), most of which are non-autonomous (i.e., miniature inverted-repeat transposable elements; e.g., the 217 bp *TcMar-N1_Ec32*, which relies on the 4.4 kb autonomous *TcMar-2_Ec32* for its transposition and exhibits 27-fold higher copy number; Fig. 1C, Supplementary Material 4). The transposase of *TcMar-2_Ec32* has acquired an N-terminal EsV-1–7 domain (Fig. 1C), which may be an uncharacterized class of zinc finger domain that has proliferated in the genomes of brown algae and are otherwise mostly known from *Nucleocytoviricota* giant viruses [47]. Although all of the autonomous *Harbinger* elements encode two tail-to-tail genes, they can be divided to two groups: elements that target ANT trinucleotides (where N is any nucleotide) and cause corresponding ANT target site duplications (TSDs; previously reported for the *Pangu* subgroup [48] and those that match the classic TNA insertion/target site duplication pattern of *Harbinger* (e.g., *Harbinger-3_Ec32*, Fig. 1C).

A remarkable feature of the *Ectocarpus* mobilome is the presence of several giant DNA transposons from the superfamilies *KDZ*, *EnSpm*, and *Sola-1*, which generally span more than 10 kb, but can exceed 30 kb. In the case of *KDZ* and *EnSpm*, the combination of their considerable lengths and high copy numbers results in these families comprising substantial proportions of the genome (1.8% and 1.1%, respectively; Fig. 1A, Table 1). Most giant transposon families feature nested insertions of unrelated TEs (e.g., *KDZ-1_Ec32*, Fig. 1C). These insertions are generally fixed among multiple copies, implying that these giant elements have continued to transpose following insertion by other TEs. Many families carry additional transcribed genes, and frequently the transposase-encoding gene is predicted to be pseudogenized, implying mobilization in *trans* via copies that can produce an active transposase. Indeed, several giant families only carry genes that have no homology to transposases and are presumably entirely non-autonomous (Supplementary Material 3), analagous to the Pack-MULE transposons of plants [49]. While most of the accessory genes are of unknown function, we identified one intriguing gene transcribed on the opposite strand to the transposase in the 21.5 kb *Sola1-1_Ec32* family (Fig. 1C). The predicted product of this gene is a 1104 aa protein featuring two domains typically associated with prokaryotes: a ParB-like DNA binding domain and a DNA methyltransferase domain predicted to produce either N4-methylcytosine or N6-methyladenine. Similar proteins have been reported in green and red algal transposons, and they may represent a novel class of transposon accessory proteins that are capable of binding and epigenetically modifying their own transposon sequence [50, 51].

### Chromosomal distribution of TEs reveals repeat enrichment on the sex chromosomes

The *Ectocarpus* v5 haploid reference genome features 27 chromosomes ranging from 4.5 to 11 Mb, including the male sex chromosome (i.e., chromosome 13 or V) that harbors the male-specific sex determining region (SDR) [33]. Although not analyzed in the above section, Liu et al. [33] also produced a gapless assembly of the female SDR (chromosome U) via sequencing of a haploid female gametophyte, which is available as part of the v5 assembly. The SDRs of the sex chromosome harbor loci involved in male (V) and female (U) sex determination [52] and do not undergo recombination [4, 14, 53]. Consequently, the SDRs are more repetitive, less gene dense, and have larger introns and lower GC content relative to the autosomes [4, 14]. The pseudoautosomal regions (PARs) flanking the SDR exhibit intermediate values to those of the SDR and autosomes for the aforementioned genomic features, despite showing a recombination rate similar to the autosomes [14, 53, 54]. To explore the evolutionary dynamics of TE accumulation on autosomes and the sex chromosomes, we analyzed the repeat content across the different genomic compartments: autosomes, PARs, and male and female sex determining regions (V and U-SDR).

**Fig. 2:**
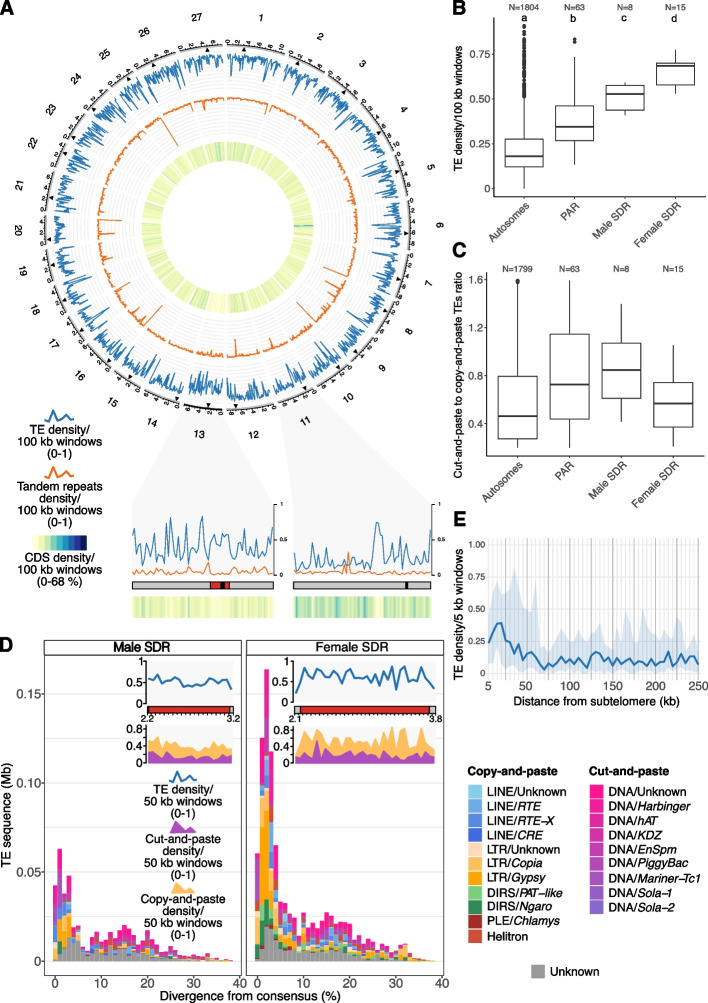
**A** Circos plot representation of the *Ectocarpus* genome. Light gray outer blocks represent chromosomes, with the sex determining region (SDR) highlighted in red on the male sex chromosome (chromosome 13). Black triangles represent the centromeric regions. Tracks represent the TE and tandem repeat densities and the inner heatmap shows CDS density. Chromosome 11 and 13 are expanded as linear representations to contrast an autosome to the sex chromosome. **B** TE density in 100-kb windows across different genomic compartments. **C** Ratio of cut-and-paste to copy-and-paste TEs in 100-kb windows. **D** TE landscapes and TE densities of the male and female SDRs. Repeat densities were calculated in 50-kb windows. **E** TE density across 5-kb non-overlapping windows extending towards the centromere from the subtelomeric satellites; median density (blue line), first and third quantiles (light blue area)

Overall, the distribution of TEs is not uniform, with total TE proportions of 22% on the autosomes, 39% on the PARs, 51% on the male SDR, and 65% on the female SDR (Supplementary Material 8: Table S2). The entire male sex chromosome harbors a significantly higher fraction of TE sequence compared to the autosomes (Kruskal–Wallis *p*-value < 2.2e − 16; Dunn’s test with Bonferroni correction shows *p*-value < 0.05 for all comparisons, except for *p* = 0.0597 in the comparison with chromosome 22; Fig. 2A, Supplementary Material 7: Fig. S3A), as previously reported [14]. Considering the SDR and PARs of the male sex chromosome separately, TEs are enriched in the SDR compared to the PARs (Fig. 2B, Wilcoxon rank sum test with continuity correction *p*-value = 1.60e − 16), and the PARs are enriched compared to the autosomes (Wilcoxon rank sum test with continuity correction *p*-value < 2.2e − 16).

As introduced above, the difference in genomic TE proportions between the PARs and autosomes is not expected given their similar recombination landscapes. Since DNA transposons are abundant in the *Ectocarpus* genome, we asked whether the mechanistic properties of DNA transposons could be responsible for the TE enrichment on the PARs. Unlike copy-and-paste elements, cut-and-paste DNA transposons exhibit biased insertion patterns termed “local hopping,” in which new insertions occur more frequently in *cis*-linked sites in the vicinity of the donor locus [55, 56]. We hypothesized that DNA transposons that have accumulated in the recombination-suppressed SDR could act as a source of local hopping events, which may have resulted in an accumulation of DNA transposons on the nearby PARs, relative to the autosomes. To test this hypothesis, we scaled the abundance of DNA transposons relative to copy-and-paste TEs (retrotransposons and Helitrons) in different genomic regions. On the autosomes, we find a copy:cut abundance ratio of 0.48 (Fig. 2C, Supplementary Material 8: Table S2). In contrast, the PARs of the male sex chromosome exhibit a significantly higher ratio of DNA transposons to copy-and-paste elements (0.71) relative to the autosomes (Wilcoxon rank sum test with continuity correction *p*-value = 2.60e − 04), as does the male SDR (0.79) (Fig. 2C). When examining TE superfamily densities per megabase, we observed that only a subset of DNA transposon superfamilies exhibit consistent enrichment on the sex chromosomes relative to the autosomes (Supplementary Material 5, Supplementary Material 7: Fig. S3B, S3C). Namely, *Tc1-Mariner*, in addition to the giant *KDZ* and *EnSpm* DNA transposons, were enriched (log₂FC > 1) in both the SDR and PAR. Taken together, these observations support the possibility that local hopping of DNA transposons, and particularly giant elements, from the SDR has contributed to the elevated DNA TE content observed in the adjacent PARs.

We next examined the male and female SDRs in more detail (Fig. 2D; Supplementary Material 8: Table S3). The female SDR is approximately twice the size of the male SDR (Supplementary Material 8: Table S2) and harbors a significantly higher abundance of TEs, with 64.5% TE content compared to 50.9% in the male SDR (Wilcoxon rank sum test with continuity correction *p*-value = 1.42e − 03) (Fig. 2B, D). Surprisingly, the ratio of cut-and-paste to copy-and-paste elements on the female SDR is 0.52, not significantly higher (Wilcoxon rank sum test with continuity correction *p*-value = 1) than the autosomes (Fig. 2C). This observation can be explained by an increased abundance of LTR retrotransposons specifically in the female SDR; LTR retrotransposons comprise 16 and 8% of the female and male SDR, respectively, and *Gypsy* elements comprise almost 10% of the female SDR. While both SDRs share similar distributions of TE divergence values for DNA transposons, the female SDR features a higher abundance of low-divergence LTR retrotransposons (Fig. 2D). TEs on the female SDR are thus significantly younger than the ones on the male SDR (Wilcoxon rank sum test with continuity correction *p*-value = 8.698e − 06), consistent with a recent expansion of LTR retrotransposons that has specifically impacted the female SDR.

We also investigated the TE distribution along autosomes. The median TE density per autosomal 100-kb window is 18.9%, although there are many outlier genomic windows that exhibit substantially higher TE densities (Fig. 2B). Indeed, the autosomal landscape is punctuated by discrete regions that are TE-rich and gene-poor; for example, a ~ 300-kb region on chromosome 6 has a TE density of 64.7% and a coding sequence (CDS) density of only 6.4% (Fig. 2A). These TE-rich islands are independent of centromeres, which were recently characterized as short regions featuring the *ECR-1* centromere-specific *Gypsy* element [33]. The centromeres span on average only 39 kb, and this highly localized enrichment does not substantially contribute to variation in TE density at a chromosome-wide scale (Fig. 2A). Conversely, we did find an enrichment of TEs towards the chromosome termini. The *Ectocarpus* subtelomeric regions are defined by the presence of a specific satellite array [33], and highly elevated abundances of tandem repeats can be observed at several chromosome termini (Fig. 2A). Immediately upstream of these subtelomere-specific satellites, we observed a localized increase in TE density extending ~ 70 kb away from the subtelomere (Fig. 2E).

Finally, although the *Ectocarpus* v5 genome is near-complete, the assembly of a small number of chromosomes did not reach the subtelomere or telomere, and there are five unplaced scaffolds (13.75 Mb in total) that likely correspond to these incomplete chromosomal termini [33]. The scaffolds are massively repetitive (as much as 70% TE sequence, Supplementary Material 8: Table S4), and although they span only 7% of the genome, the scaffolds constitute 18% of the total genomic TE content. These gene-poor, TE-rich regions likely correspond to large repetitive islands and putatively represent extreme cases of the TE-enrichment of chromosomal termini reported above.

### Distribution and characteristics of TE insertions relative to genic sequence

The consequences of transposon insertions on the host genome often depends on the type of the element and on its insertion site [57]. In particular, TEs can impact the regulation of nearby genes via multiple complex interactions [10]. Therefore, we examined the local distance-distribution of TEs relative to genes focusing on the 27 assembled chromosomes representing the haploid male genome.

**Fig. 3:**
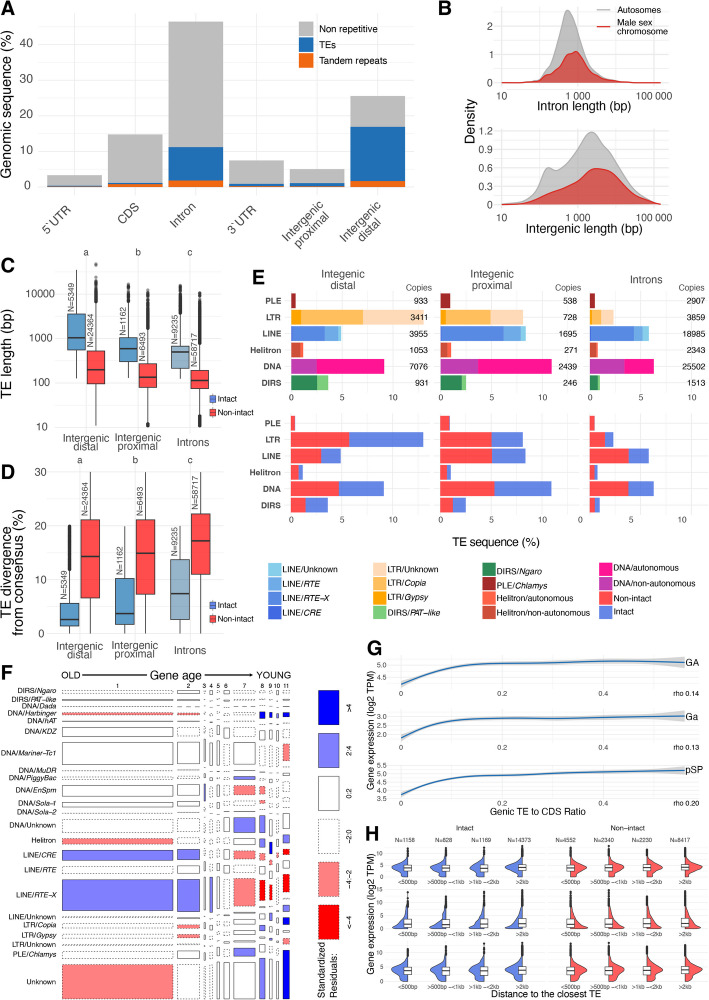
**A** Density of TE bases by genomic site class. The blue and orange bars represent the proportion of sequence occupied by TEs and tandem repeats, respectively; gray bars represent the proportion of non-repetitive sequence. **B** Density plots showing the length distribution of introns (upper panel) and intergenic regions (lower panel) in autosomes and on the male sex chromosome. **C** Intact (blue) and non-intact (red) TE lengths by site class. **D** Divergence from consensus for intact (blue) and non-intact (red) TE copies; TEs with divergences > 30% were excluded. **E** Density of different TE classifications by site class. The bars are colored by order and superfamily in the upper panel (or autonomous/nonautonomous status) and by intact status in the lower panel. Note that the densities differ from those in panel **A** since an entire TE copy was considered “proximal” if any of its sequence was within 500 bp of a gene. **F** Mosaic plot showing the relationship between intronic TEs of different superfamilies and the age of the genes in which they are inserted; the gene age is shown as numbers from 1 to 11 where 1 are the most evolutionary ancient genes and 11 are *Ectocarpus*-specific genes. **G** Expression of genes relative to the ratio of CDS to total genic TE content, for different life stages. GA: gametophyte, Ga: gametes, pSp (parthenosporophyte). **H** Gene expression relative to the distance to the closest intact or non-intact TE

Intergenic regions contain the highest density of TEs at 52.9%, followed by 20.1% of intronic sequence, 7.0% of 5’ UTRs, 8.8% of 3’ UTRs, and 2.4% of CDS (Fig. 3A, Supplementary Material 8: Table S5). Conversely, tandem repeats exhibit a relatively even distribution across the genome (Fig. 3A, Supplementary Material 8: Table S5). The *Ectocarpus* genome is generally highly compact with regard to intergenic space, with a median intergenic length of 1302 bp on the autosomes, rising to 2853 bp on the male sex chromosome (Fig. 3B). To explore the difference between intergenic sequence immediately adjacent to genes and that far from genes, we divided intergenic regions into gene “proximal” (sequences within 500 bp of a gene) and “distal” (sequences > 500 bp from a gene) categories. E This 500 bp threshold from both flanks of neighboring genes captures the median intergenic tract length. Proximal intergenic regions exhibited a substantially lower TE density (17.8%) relative to distal intergenic sequences (59.6%) (Fig. 3A). Thus, genes generally do not harbor a high density of TEs in their immediate flanking sequence, and the high overall density of intergenic TEs can largely be explained by longer repeat-rich regions that are generally distant from genes.

The *Ectocarpus* genome, and indeed most brown algal genomes [2], has a very high intronic content (~ 46% of the genome) [58]. Furthermore, the *Ectocarpus* introns are far larger than model species with similarly compact genomes, where intron lengths are generally tightly distributed with a peak of 60–110 bp (e.g., *Arabidopsis thaliana* and *Drosophila melanogaster*) [59]. The *Ectocarpus* introns have a median length of 524 bp on the autosomes and 765 bp on the sex chromosome (Fig. 3B). These intron features, combined with the high percentage of intronic TEs, prompted us to investigate the relative characteristics of intronic and intergenic TEs. TEs inserted within introns are shorter (Kruskal–Wallis rank sum test *p*-value < 2.2e − 16) and more divergent from their consensus sequences (Kruskal–Wallis rank sum test *p*-value < 2.2e − 16) compared to intergenic TEs (Supplementary Material 8: Table S6). These differences are consistent for both intact and non-intact elements (Fig. 3C, D), where intact elements are defined as copies that span > 80% of the consensus length and are < 20% divergent from the consensus (i.e., copies that may retain the capacity to transpose).

The intergenic and intronic populations of TEs also differ in terms of their TE compositions. Intergenic proximal and intronic regions are predominantly populated by DNA transposons and LINEs, whereas LTR retrotransposons contribute the majority of TE sequence to intergenic distal regions (Fig. 3E, Supplementary Material 8: Table S7). DNA transposons consistently exhibit the highest copy numbers relative to the other subclasses, and autonomous elements dominate the total abundance in intergenic sequence but not introns, where the abundance of autonomous and non-autonomous DNA transposons is approximately equal (Fig. 3E; Supplementary Material 8: Table S7). Across all subclasses, the proportion of intact elements is generally highest in intergenic distal sequences, followed by intergenic proximal sequences, and introns (Fig. 3E). These results suggest that the shorter length of intronic TEs can be explained by both a higher density of shorter insertions (non-autonomous DNA transposons, LINEs that are frequently 5’ truncated and include very short abundant non-autonomous families, see Fig. 1C) and a greater abundance of older, degraded TE copies. Conversely, LTR elements, which insert as full-length copies and have lengths ranging between 3.9 and 14.5 kb in *Ectocarpus*, dominate the intergenic distal regions together with autonomous DNA transposons, which include the giant families discussed above.

Brown algal genomes feature genes that span an extensive range of evolutionary ages, with many genes taxonomically restricted to the entire clade or to specific brown algal lineages [2, 4, 60]. We next tested whether there was any difference in the properties of intronic TE insertions among genes of different evolutionary ages. We found that LINEs tend to be enriched within evolutionarily old genes (phylostratum rank 1), assessed with genomic phylostratigraphy [60], whereas *Harbinger* DNA transposons and LTR retrotransposons are over-represented in younger genes that emerged after the evolution of the Ectocarpales (phylostratum rank 8) (Fig. 3F). Notably, elements that can be very large, specifically LTR retrotransposons and Helitrons, are over-represented within *Ectocarpus*-specific genes (phylostratum rank 11) (Fig. 3F). Together with the above observations that intronic TEs are generally shorter and more degraded relative to intergenic TEs, these results suggest that selection may play a major role in shaping the composition of intronic TE sequences, with longer TEs only tolerated in evolutionarily young genes that may be evolving under weaker selection.

Finally, we explored the effects of TE insertions on gene expression. Since intronic TEs are ubiquitous in *Ectocarpus* (81% of genes feature at least one genic insertion), we tested whether the abundance of intronic TEs is associated with gene expression levels. To quantify intronic TE abundance per gene, we calculated the ratio of genic TE nucleotides to CDS nucleotides. We found a very weak positive monotonic correlation between intronic TE abundance and gene expression (rho 0.14 in the gametophyte (GA) stage, rho 0.13 in the gametes, rho 0.20 in the parthenosporophytes (pSP). While the *p*-values are highly significant (*p*-value < 2.2e–16), this is likely due to the large sample size, and the relationship between TE content and gene expression is rather weak (Fig. 3G), suggesting that genes can be highly expressed despite containing substantial intronic TE densities. The absence of any major effect of intronic TE content on transcript abundance was consistent across three distinct life stages, the GA, gametes, and pSP. Considering the distance to the nearest intergenic TE, we found no effect of the vicinity of either intact or non-intact TEs on gene expression (Fig. 3H). Furthermore, 82% of genes had no intact TE within 2 kb, although 52% of genes did have at least one non-intact TE in these regions (Fig. 3H). This result suggests that proximal TE insertions are selected against in the *Ectocarpus* genome and is consistent with the presence of large gene-poor TE-rich islands that harbor mostly intact TEs.

### Small RNAs and non-canonical histone modifications are associated with potentially active TEs

**Fig. 4:**
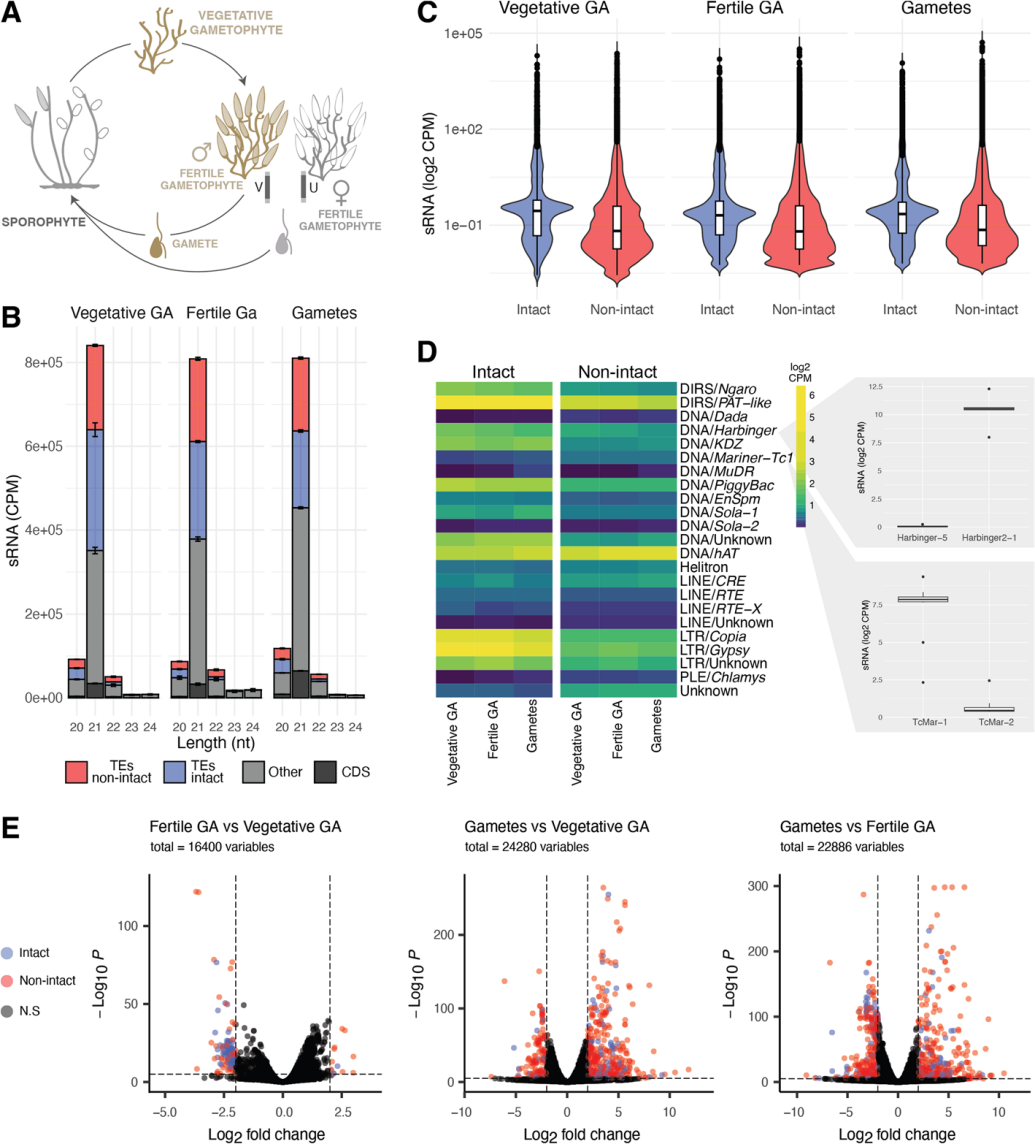
**A** Life cycle of a male *Ectocarpus*. The colored life stages (vegetative gametophyte, fertile gametophyte, gametes) represent those sampled for sRNA sequencing in this study. **B** Size distribution and genomic feature annotation of sRNAs. Error bars represent SD of the mean of the biological replicates. **C** sRNA accumulation (CPM) on intact (blue) and non-intact (red) TE copies across the life stages. **D** sRNA accumulation (CPM) by TE superfamily. The zoomout panels show sRNA accumulation (CPM) differences between specific families from the *Tc1-Mariner* and *Harbinger* superfamilies. **E** Volcano plots illustrating TEs with differential sRNA expression throughout the *Ectocarpus* life cycle. Intact TEs are represented by blue dots, and non-intact TEs by red dots

Both small RNAs and H3K79me2 have been shown to associate with TEs in Ectocarpus [27, 31]. We therefore asked whether these associations play a role in the regulation of TEs. We performed sRNA-seq across three different stages of the life cycle, namely the vegetative and fertile gametophyte and gametes of the Ectocarpus sp. 7 male reference strain Ec32 (Fig. 4A). Consistent with [27] and [32], we recovered a peak in sRNA abundance at 21 nt in all three life stages (Fig. 4B) and a strong uracil (U) bias at the 5’ end (Supplementary Material 7: Fig. S4A), consistent with the presence of a single Argonaute (AGO) protein encoded in the genome. Across all three developmental stages, the abundance of sRNAs that mapped to TEs was substantially higher than those mapping to CDS (49,647.51 CPM average versus 10,640.45, Fig. 4B). A substantial proportion of sRNAs also mapped to other regions of the genome, which likely includes miRNA loci [32]. We found that CDS-mapping sRNA reads of 24 nt or longer were almost entirely in the same sense as the associated mRNA (Supplementary Material 7: Fig. S4B, S4C), consistent with degradation products. Conversely, on both TEs and CDS, sRNAs < 24 nt exhibited relatively equal proportions of sense and antisense mapping, and only 1% (0.4% in gametes) of TE-associated reads were 24 nt or longer (Supplementary Material 7: Fig. S4D, S4E, Supplementary Material 8: Table S8). We therefore focused on sRNAs of 20–24 nt for our analyses.

sRNAs overlap with 28.1% of TEs across all developmental stages (CPM > 0, Supplementary Material 6). Noticeably, the percentage of TEs associated with sRNA in all life stages increases to 64.7% when considering intact TEs exclusively and falls to 21.8% for non-intact TEs (Supplementary Material 8: Table S9). Furthermore, sRNA accumulation is significantly higher on intact TEs compared to non-intact TEs (Wilcoxon rank sum test with continuity correction *p*-value < 2.2e–16) (Fig. 4C). This enrichment towards intact TEs holds for all developmental stages and across TE superfamilies (Fig. 4C, D). Therefore, sRNAs in *Ectocarpus* are preferentially associated with intact, potentially active, transposons.

For both intact and non-intact TEs, sRNA accumulation varies substantially across different TE categories (Fig. 4D). For example, sRNA CPM values are consistently higher for LTR retrotransposons relative to LINEs; however, due to the challenges of sRNA normalization in terms of TE size, the result should be interpreted with caution. For other subclasses, we observed considerable differences in sRNA accumulation between superfamilies, e.g., *Ngaro* DIRS elements exhibit lower sRNA CPMs (1.81 log2CPM) than *PAT-*like DIRS (6.38 log2CPM), as do *Harbinger* DNA transposons (1.67 log2CPM) relative to *Mariner-Tc1* DNA transposons (0.33 log2CPM). Most strikingly, we found that specific families within the same superfamily could differ drastically in their association with sRNAs. For example, within the *Mariner-Tc1* superfamily, the autonomous family *TcMar-1_Ec32* exhibits 12-fold higher sRNA log2CPMs than *TcMar-2_Ec32* (Fig. 4D), and two *Harbinger* families differ similarly (Fig. 4D). The association between sRNAs and TEs thus appears to be dynamic and clearly non-random, suggesting a role for sRNA in the regulation of specific TE families.

To further assess the dynamics of TE regulation across the life cycle of *Ectocarpus*, we performed differential sRNA accumulation analysis between the three sampled life stages (Fig. 4A). Most TE copies (> 98%) exhibited no significant differential sRNA accumulation between the life stages, suggesting that TE regulation via sRNAs is stably maintained. However, 1.2 and 1.4% of the total TE copies showed differential sRNA accumulation between vegetative gametophytes and gametes, and fertile gametophyte and gametes, respectively (Fig. 4E, Supplementary Material 7: Fig. S5A, S5B; Supplementary Material 8: Table S10, S11). The changes in sRNA association with TEs during this transition were not strongly biased towards either upregulation or downregulation, suggesting a general reconfiguration of TE-associated sRNA accumulation in gametes (Supplementary Material 8: Table S10, S11). Non-intact TEs were significantly more likely to exhibit differential sRNA accumulation compared to intact elements in both gametophyte-to-gamete comparisons (Supplementary Material 8: Table S12; chi-square test, *p* < 0.00001). This pattern may reflect the importance of maintaining silencing mechanisms for intact TEs in the new generation.

A small subset of 166 TE copies showed differential sRNA accumulation at the transition to fertility (i.e., between vegetative and fertile gametophytes); however, we noticed that this transition featured substantially more downregulated TEs, a significant proportion of which were intact elements (61.2% intact, relative to less than 30% in other transitions) (Fig. 4D, Supplementary Material 7: Fig. S5A). Moreover, 92.5% of these downregulated intact TEs belong to a single family of non-autonomous *Harbinger* DNA transposons (*Harbinger-N4_Ec32*, Supplementary Material 8: Table S13). These *Harbinger* elements are not associated with any significant changes in sRNA accumulation between any other life stages (Supplementary Material 7: Fig. S5B), potentially suggesting that the transition to fertility is associated with a highly specific shift in sRNA abundance in this family. However, since sRNAs almost invariably multimap to different copies of the same TE family, we cannot rule out an alternative explanation where a single copy of *Harbinger-N4_Ec32* may be inserted at a genomic locus that undergoes differential sRNA regulation.

**Fig. 5:**
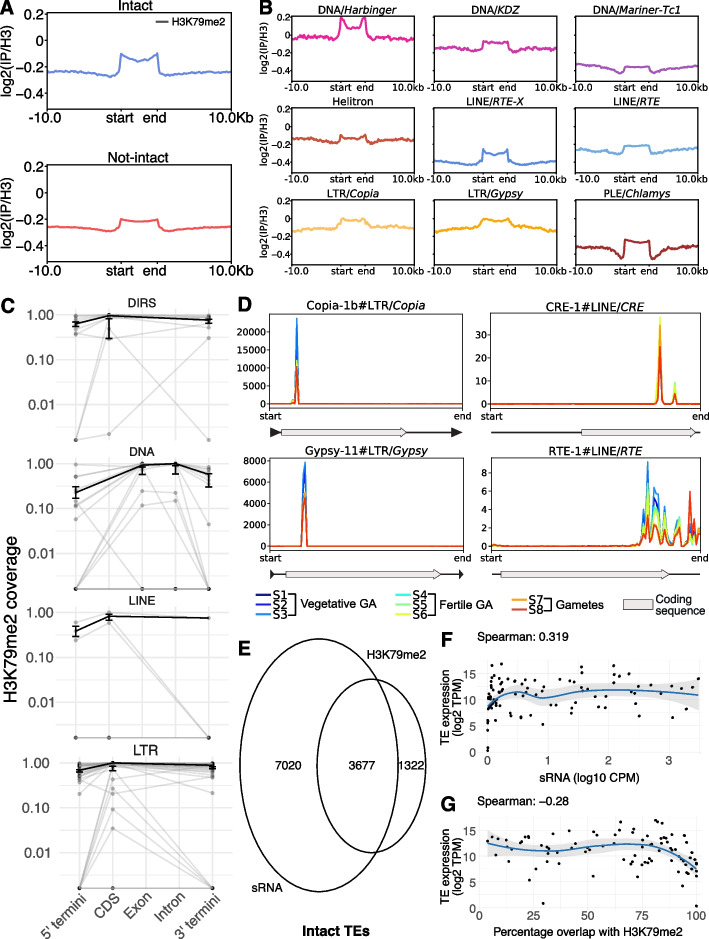
**A** H3K79me2 (normalized on H3) accumulation profiles on intact and non-intact elements. Start and end indicate the TE body, with 10-kb flanking regions. **B** H3K79me2 (normalized on H3) accumulation profiles on TE superfamilies. Start and end indicate the TE body, with 10-kb flanking regions. **C** H3K79me2 coverage over TE features. Each gray dot represents the mean coverage value, across 8 biological replicates, for each TE family consensus sequence. Gray lines connect dots belonging to the same TE family. The black line represents the median coverage value for each TE feature, with SD, within the TE subgroup represented. **D** sRNA reads distribution over TE body. Each line represents a biological replicate. **E** Venn diagram showing the intersect between intact TE copies associated with sRNA and H3K79me2. **F** Scatter plot and fitted curve (Loess regression) showing the correlation between sRNA abundance (CPM) on intact TE copies and TE gene expression. Each point represents a TE family. **G** Scatter plot and fitted curve (Loess regression) showing the correlation between the average proportion of intact TE body overlapped by H3K79me2 peaks and the TE gene expression

We next assessed the relationship between H3K79me2 and TEs. We identified H3K79me2 peaks in the *Ectocarpus* v5 genome (Methods), and we found that 30.2% of intact TEs overlap with an H3K79me2 peak, relative to 23.1% of non-intact TEs. Furthermore, H3K79me2 signal was consistently higher within the TE body relative to the flanking regions, with a more pronounced increase in signal for intact TEs (Fig. 5A). Among TE superfamilies, *Harbinger* DNA transposons exhibited the most substantial enrichment in TE body H3K79me2 signal (Fig. 5B, Supplementary Material 7: Fig. S6A). Note that many TEs are in close proximity to other TEs, which reduces the relative signal of TE body to flanking sequence.

To explore the potential interplay between sRNA and H3K79me2 in TE regulation, we mapped both ChIP-Seq and sRNA reads to TE consensus sequences (Methods). Specifically, we split autonomous TEs into their left and right termini and genic sequence; for retrotransposons, we considered CDS, for DNA transposons exons and introns. We found that DIRS (*p* = 0.024), DNA transposons (*p* = 0.023), and LTR retrotransposons (*p* = 5.9 × 10⁻⁷) exhibited significant differences in H3K79me2 coverage across annotated features. In particular, DIRS and LTR retrotransposons showed significantly higher coverage over internal coding regions compared to terminal regions, especially the 5′ end (Fig. 5C, Supplementary Material 8: Table S14). For DNA transposons, although the overall *p*-value suggested a trend towards greater coverage over internal features (exons, introns), none of the pairwise comparisons remained significant after multiple testing correction, indicating a weaker or more variable pattern. Other TE groups did not show statistically significant differences. Nonetheless, individual TE families often exhibited distinct coverage profiles, with internal regions frequently showing elevated H3K79me2 coverage.

These observations led us to ask whether H3K79me2 preferentially targets TE-encoded genes. Supporting this idea, we found that autonomous TEs, which typically contain intact coding sequences, exhibited significantly higher H3K79me2 coverage than non-autonomous TEs (*p* = 0.034; Supplementary Material 8: Table S15). Together, these results indicate that H3K79me2 may play a role in the transcriptional repression of TE genes.

Similarly, sRNA accumulation profiles were highly variable across TE sequences. Within superfamilies, sRNA peaks were not significantly associated with specific annotated TE features (Fig. 5D, Supplementary Material 7: Fig. S6B, S6C). However, sRNAs did not accumulate uniformly along the TE body, but tended to concentrate in localized regions, consistently across life stages. For example, both LTR/*Copia* and LTR/*Gypsy* elements accumulate sRNA preferentially towards the 5’ end of the TE body; on the contrary LINEs typically exhibit sRNA accumulation towards the 3’ end (Fig. 5D, Supplementary Material 7: Fig. S6B).

Considering sRNA and H3K79me2 collectively, only 8.6% of TE copies are associated with both sRNA mapping and a H3K79me2 peak (Supplementary Material 7: Fig. S7A). This number increases to 22.2% of intact TEs, and strikingly, 73.6% of intact TEs that intersect an H3K79me2 peak also exhibit sRNA mapping (Fig. 5E). Thus, most intact TEs associated with H3K79me2 are also associated with sRNAs, whereas the majority of intact TEs associated with sRNAs are not associated with H3K79me2.

We next investigated the differences between the sRNA population associated with intact TEs that intersect an H3K79me2 peak and the set associated with TEs that do not intersect with an H3K79me2 peak. The length distribution of sRNA in the two populations confirms the peak at 21nt (Supplementary Material 7: Fig. S7B, S7C), with only small differences in strand preference: the 21nt sRNA associated with TEs that intersect an H3K79me2 peak are equally sense and antisense, with antisense sRNA becoming more abundant among 23 and 24 nt sRNA (Supplementary Material 7: Fig. S7B). In contrast, sRNAs associated with TEs that do not overlap with the histone mark present a higher abundance of antisense 21 nt sRNA (Supplementary Material 7: Fig. S7C). This observation potentially highlights the importance of post-transcriptional gene silencing (PTGS) in TEs that lack H3K79me2. Alternatively, it may suggest that sense sRNAs contribute to the deposition of H3K79me2. Furthermore, we observed no difference in 5′ nucleotide bias between the two sRNA populations; both show a strong preference for uracil (U) at the 5′ end (Supplementary Material 7: Fig. S7D, S7E; Supplementary Material 8: Table S16). Regarding the distribution of intact TEs relative to genes, the majority of both intergenic and intronic TE copies lack H3K79me2 marks (59 and 72%, respectively; Supplementary Material 8: Table S17). Nonetheless, intact intergenic TEs are more likely to associate with an H3K79me2 peak than intronic copies (chi-square *p*-value < 0.00001). These results suggest that H3K79me2-marked TEs tend to be located further from genes, potentially reflecting a mechanism to prevent unintended silencing of nearby genes due to the broad distribution of H3K79me2 peaks. Alternatively, the lower proportion of marked TEs within introns may reflect the reduced presence of autonomous TEs in these regions, as H3K79me2 appears to preferentially target TEs encoding transposition-related proteins.

Finally, we explored the relationships between TE gene expression across the same life stages, and sRNA and H3K79me2 association. Restricting the analysis to manually curated TE families that encode genes, the nine most highly expressed TE families are all *Copia* LTR retrotransposons (Supplementary Material 7: Fig. S7C, Supplementary Material 8: Table S18), which dominate the most expressed TE families across all life stages (Supplementary Material 7: Fig. S7D, Supplementary Material 8: Table S18). In total, 97.9% of these gene-encoding families exhibited expression of at least 1 TPM (transcripts per million) consistently across life stages, suggesting broad transcriptional activity for intact TEs in *Ectocarpus*. We found a weak positive correlation (Spearman’s rho = 0.319) between TE gene expression and sRNA abundance on intact copies, suggesting that TEs associated with high sRNA abundance can still be highly expressed (Fig. 5F). Conversely, we observed a weak negative correlation (Spearman’s rho = − 0.28) between TE gene expression and the average percentage of intact TEs overlapped by H3K79me2 peaks (Fig. 5G). In particular, the curve applied to highlight the trend between variables (Loess Regression) shows a pronounced decline in TE expression when more than 75% of the TE body intersects with H3K79me2 peaks (Fig. 5G). Therefore, coverage of a high proportion of the TE body by H3K79me2 is associated with reduced transcriptional activity.

## Discussion

### The diverse mobilome of *Ectocarpus* and TE-enrichment on the sex chromosome

Despite occupying only 28% of its genome, *Ectocarpus* features a high diversity of TEs, harboring nearly 340 families from six subclasses and 19 superfamilies. Furthermore, more than half of the cumulative TE sequence in the genome belongs to copies that exhibit less than 5% divergence from their family consensus sequence, suggesting recent or potentially ongoing transposition. This is supported by our observation that ~ 98% of curated autonomous TE families are expressed consistently across life stages, potentially leading to the production of transposon proteins. Population genomics or experimental data will be required to demonstrate active transposition in *Ectocarpus*. The skew of the TE landscape towards younger elements also implies that the *Ectocarpus* genome is under selection to maintain compactness, with inactive and degrading TEs being purged from the genome. Although detailed analyses of TE landscapes and TE diversity have not been performed for other brown algae, their greater genome sizes and repeat densities [2] suggest that selection against TE accumulation may be weaker in several species, many of which are morphologically more complex and would be expected to have lower effective population sizes.

The substantial diversity of *Ectocarpus* TEs highlights the benefits of studying taxonomically diverse genomes. For example, our annotation of *Gypsy* LTRs increases the number and diversity of known fusions between integrases and chromatin-reader domains, and revealed acquisitions of NADAR domains by diverse retrotransposons. Whether these retrotransposons may facilitate this diversification process through extrachromosomal recombination as suggested for other species [61] will be an exciting area for future research. Notably, DNA transposons are the most abundant and diverse TE subclass in *Ectocarpus*, spanning almost 8% of the genome. This proportion is likely to be an underestimation, as most of the remaining unknown TEs are expected to be DNA elements. Automated classification of DNA transposons is highly challenging [35]; for autonomous families, nucleotide-to-protein homology searches are complicated by the abundance of introns in transposase genes and low sequence conservation of DD(E/D) transposases, whereas nonautonomous families are often very short and lack obvious structural characteristics. Although DNA transposons undergo cut-and-paste transposition, they can increase in copy number during certain transposition events [9, 62], and the relative abundance of retrotransposons and DNA transposons differs substantially between eukaryotes. For example, although TEs comprise approximately the same genomic space, in *C. elegans* DNA transposons account for more than 70% of the total TE content [63] whereas in *A. thaliana* this figure is ~ 27% [64] and in *D. melanogaster* only ~ 7% [65]. DNA transposons are also the dominant subclass in many, but not all, fish genomes, whereas they are absent from most mammalian genomes [66]. Furthermore, TE horizontal transfer events in vertebrates overwhelmingly occur among fish [66]. Like many fish, brown algae undergo external fertilization in aquatic environments where opportunities for horizontal transfer may be increased, and it would be interesting to address the evolutionary origin of brown algal DNA transposons in future work.

An additional contributing factor to the abundance of DNA transposons in the *Ectocarpus* genome is the presence of several giant elements, specifically from the *KDZ*, *EnSpm*, and *Sola-1* superfamilies, which have maximum lengths ranging from 15.2 kb (*EnSpm*) to 30.3 kb (*KDZ*). *KDZ* elements encode complex transposases that can exceed 1300 amino acids [67], frequently carry accessory genes in fungi [68], and are among the largest DNA transposons reported. Active transposition of a 31.7 kb *KDZ* element was observed in the green alga *Chlamydomonas reinhardtii* [69], and giant *EnSpm* elements carrying multiple accessory genes have been reported in red algae [51]. The evolution of giant TEs is thought to be limited due to the risk of ectopic recombination occurring between copies, which would be strongly selected against by the host [70]. *Ectocarpus*, along with many other taxa associated with giant TEs (fungi, green and red algae), spends a substantial portion of its life cycle in the haploid stage [7]. Although DNA repair mechanisms in *Ectocarpus* remain uncharacterized, a potentially low frequency of homologous recombination in the haploid stage could help explain the evolution of giant transposable elements in this species.

Consistent with previous studies, we found that TEs are enriched on the *Ectocarpus* sex chromosomes [4, 53]. Notably, this enrichment is not restricted to the non-recombining SDR but also extends into the PARs, despite PARs recombining at rates comparable to autosomes [4, 53]. This pattern contrasts with observations in other organisms with haploid chromosomal sex or mating type determination; for example, *Microbotryum* fungi show no TE enrichment in their recombining PARs [71]. We hypothesize that the high abundance of DNA transposons in the *Ectocarpus* genome may underlie this pattern. In this model, DNA transposons would initially accumulate in the non-recombining sex-determining region (SDR), along with other repetitive elements. Due to the known tendency of DNA transposons to insert near their donor copies (a phenomenon known as “local hopping”; [55]), the SDR could act as a source of transposition events, leading to increased insertion rates in the adjacent PARs. Over time, the resulting accumulation of DNA transposons in the PARs could create a reservoir of relatively neutral sequence, facilitating the secondary accumulation of other TEs that might otherwise be subject to stronger purifying selection. Although this hypothesis is difficult to test directly, we do observe a significant enrichment of DNA transposons (relative to copy-and-paste elements) in the *Ectocarpus* PARs. In contrast, *Microbotryum* genomes contain relatively few DNA transposons and are instead dominated by LTR retrotransposons [71], consistent with the absence of TE enrichment in their recombining PARs.

The *Ectocarpus* female SDR contains a distinct set of TEs compared to the male SDR. Similar patterns of sex-chromosome TE differences have been reported in other species and attributed to sex specific activity of certain TEs or differences in epigenetic silencing between sexes. For example, in *Silene*, the Y chromosome shows different TE content compared to autosomes [72] and in the dioecious plant *Rumex acetosa* the X and Y chromosomes exhibit differing TE distributions [73]. In animals, sexually dimorphic TE expression in the germline has been linked to elevated TE activity in males, which undergo more mitotic divisions, a trend consistent with the positive correlation between TE activity and mitotic rate [74]. However, *Ectocarpus* has minimal morphological and developmental differences between male and female gametophytes. Thus, sex-specific silencing or expression is unlikely to account for the observed TE differences between male and female SDRs. A more plausible explanation is that, due to high population polymorphism, the female lineage sampled in this study randomly acquired a different set of TEs in the SDR, which then became fixed due to the lack of recombination in this region. Population genomics approaches will be required to test whether differences between the *Ectocarpus* male and female SDRs hold across the species.

### *Ectocarpus* has high intronic TE density, but intact TEs are primarily located far from genes

*Ectocarpus* exhibits two major TE populations within the genome: intergenic TEs (60.8% of TE bases) and intronic TEs (34.1%). Introns comprise a major fraction of the *Ectocarpus* genome and are substantially longer than introns in many model organisms with comparable genome sizes. Indeed, *Ectocarpus* introns feature exonic-splice enhancers similar to those of mammals, which may be considered an adaptation to the intron-richness of the genome [58]. We found no strong relationship between intronic TE content and gene expression, suggesting that *Ectocarpus* can tolerate a moderately high intronic TE content with little impact on gene expression, similar to several plant species [75]. Nonetheless, we do find evidence for selection against TEs in introns; intronic TEs are shorter, more degraded, and less frequently intact relative to intergenic TEs, and dominated by TE families that are naturally shorter (e.g., LINEs and LINE-dependent nonautonomous elements, nonautonomous DNA transposons). Indeed, insertions of the longest TE families, LTRs and Helitrons, are only preferentially found in *Ectocarpus*-specific genes, which may be evolving under much weaker selection relative to evolutionarily ancient genes.

Intergenic TEs in *Ectocarpus* show distinct characteristics depending on their proximity to genes. TE insertions adjacent to genes are more frequently shorter and more degraded, and most genes do not feature an intact TE of any length within 2 kb. Hence, the greatest abundance of TEs in the *Ectocarpus* genome, and especially longer elements such as LTR retrotransposons and autonomous DNA transposons, are concentrated in gene-poor repetitive islands that are not associated with the centromeres. Many genomes feature similar TE-rich islands, for example in some fungi [76] and insects [77], and these regions can be associated with rapid evolution and extensive structural polymorphism at the population level [78]. The paucity of intact TEs near to genes may also explain the absence of any obvious relationship between gene expression and distance to the nearest TE, which, for example, may be expected if repressive histone marks associated with the TE were to influence expression of the neighboring gene. Assessment of the effect of rare TE insertions on gene expression across an *Ectocarpus* population would be one avenue to address this question [79].

### Regulation and repression of transposable elements in *Ectocarpus*

Host silencing and regulation of TEs is highly complex and frequently involves sRNAs, DNA, and histone modifications, as well as crosstalk between these mechanisms [80, 81]. In *Ectocarpus*, which notably lacks cytosine methylation, the mechanisms underlying effective TEs control remain largely unknown, and this is particularly striking given the high density, diversity, and transcriptional activity of intact and potentially active elements.

Our results reveal a clear enrichment for sRNAs towards intact TEs. Furthermore, sRNAs association is not uniform across different TEs; TE families within the same superfamily (e.g., *Tc1-Mariner* DNA transposons) can exhibit markedly different sRNA targeting. These findings suggest that sRNAs do not simply target recent, potentially active TE insertions, but rather a specific subset of elements. We also show that sRNAs show consistent, localized patterns along TE bodies, supporting the hypothesis of a functional role in TE silencing. 21-nt sRNAs are typically linked to post-transcriptional gene silencing (PTGS) in other systems such as plants, where sRNAs of different lengths are associated with distinct silencing pathways [82]. In *Ectocarpus*, given the lack of other characteristic sRNA classes and a consistent association between sRNA and specific TE features, it remains unclear whether these sRNAs mediate PTGS, participate in transcriptional silencing, or play multiple roles depending on the context.

Approximately a third of intact TEs are also associated with H3K79me2, which has been linked with decreased transcript abundance in *Ectocarpus* [29]. H3K79me2 peaks are typically localized within the TE gene body, suggesting a role in transcriptional repression. Notably, most intact TEs that are associated with H3K79me2 enrichment show sRNA accumulation, whereas most intact TEs that lack H3K79me2 are still associated with sRNAs. This could imply that there is some independence between these putative silencing mechanisms, or that sRNAs play multiple roles in TE control. Indeed, while we did find a weak repressive effect of H3K79me2 on TE gene expression when the mark is present across most of the TE body (> 75% of TE copy), there was no clear relationship between sRNA abundance and TE gene expression. sRNAs are known to play multiple roles in TE silencing [83]. Beyond their well-characterized involvement in post-transcriptional gene silencing (PTGS) through the cleavage of target mRNAs [84, 85], sRNAs are also implicated in transcriptional silencing pathways. In animals lacking cytosine methylation, such as *C. elegans*, sRNAs have been demonstrated to directly initiate the deposition of repressive histone marks on TEs [86, 87]. Similarly, in plants, sRNAs are involved in pathways that facilitate the establishment of repressive histone modifications, such as H3K9me2 at specific TE loci and developmental stages including *Arabidopsis* embryos [88]. Although the interaction between histone PTMs and sRNAs remains elusive in brown algae, the observation that more than 70% of intact H3K79me2-associated TEs also associate with sRNAs suggests that a pathway similar to that observed in other organisms may exist. However, given the prevalence of TEs associated with sRNAs but lacking H3K79me2, it is unlikely that sRNAs function exclusively to drive histone modifications. Disentangling these possibilities will require experimental tools currently lacking in *Ectocarpus*. For instance, degradome sequencing could test whether sRNAs direct mRNA cleavage, while loss-of-function mutants for AGO1 or histone methyltransferases could determine the functional consequences of sRNA and H3K79me2 loss.

The association between sRNA and TEs remains largely stable throughout the life cycle of *Ectocarpus*, with only a small fraction of TE copies (< 2%) showing differential sRNA association during the developmental transitions studied. Despite this overall stability, some differences in sRNA accumulation are observed particularly between the gametophyte stage (both vegetative and fertile) and the gametes. These differences are pronounced over non-intact TEs, possibly reflecting their reduced silencing priority or a role in reproductive reprogramming as documented in other organisms [81, 89]. The observed differences are not biased towards increased or decreased sRNA accumulation, as some TE copies exhibit higher sRNA association in gametes compared to the gametophyte stage, while others show the opposite trend.

Our sRNA sampling spanned the developmental transition from immature to fertile gametophytes. In *Ectocarpus*, any vegetative gametophyte cell has the potential to develop into reproductive structures where gametes are formed. Therefore, a stable sRNA landscape, particularly targeting potentially active TEs, may play a critical role in safeguarding genome integrity during this sensitive developmental window.

## Conclusions

**Fig. 6:**
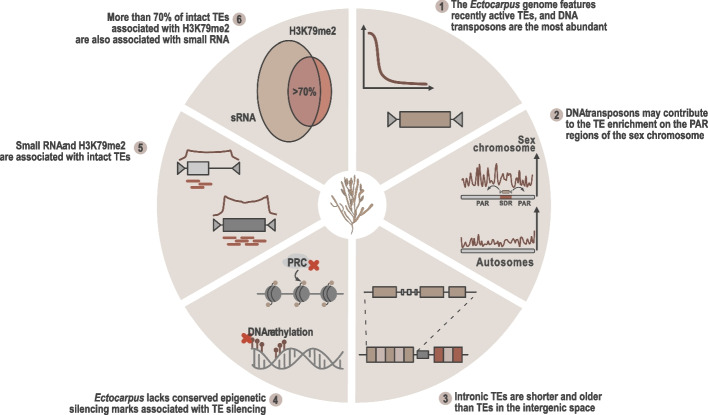
Schematic illustration of the key findings on the *Ectocarpus* mobilome

Our study reveals a rich genetic diversity of the *Ectocarpus* mobilome and uncovers a complex, multilayered regulatory landscape shaped by small RNAs and chromatin modifications (Fig. 6). These mechanisms operate in the absence of canonical epigenetic silencing pathways found in animals and plants, suggesting the presence of unique, lineage-specific mechanisms for transposable element control in *Ectocarpus* that remain to be uncovered. This work provides new insights into TE regulation in non-model eukaryotes and expands our understanding of epigenetic innovation across lineages.

## Methods

### De novo annotation and manual curation of transposable elements

Manual curation of the most abundant *Ectocarpus* TE families was performed following the methodology of [90]. Briefly, preliminary repeat models were generated by running RepeatModeler v2.0.5 [91] on the v5 reference genome using the “LTRStruct” model [92, 93]. The abundance of each automated repeat model in the genome was then estimated by passing the output of RepeatModeler to RepeatMasker v4.0.9 (https://www.repeatmasker.org/). Repeat models that contributed more than 100 kb to the genome were then selected for curation. Individual copies of each repeat were retrieved from the genome using blastn v2.9.0 (“-evalue 10e-10”) [94] extended to include flanking sequence of 2 kb, and aligned using mafft v7.453 (“E-INS-i” setting) [95]. Alignments were then viewed manually and full-length transposons were identified where possible by identifying regions where sequence from multiple independent aligned copies transitioned into unalignable flanking sequence (with iteration of the previous steps performed if the initial extension and alignment did not reach both TE termini). Consensus sequences corresponding to individual full-length families were then produced using the AdvancedConsensusMaker tool (hiv.lanl.gov). For transposons carrying genes, putative protein sequences were predicted by searching for the longest ORFs (intron-less genes) or by using RNA-seq alignments (see below) to curate gene models (intron-containing genes) in the IGV genome browser v2.15.4 [96]. A small number of lower abundance elements were also subjected to manual curation, including several autonomous DNA transposons (to aid classification of associated and more abundant nonautonomous elements) and all families that had previously been manually curated for *Ectocarpus* and made available at the Repbase repository (version 29.02) [97]. Classification was performed based on established characteristics of TE superfamilies, including homology to curated transposases, terminal structures and motifs (LTRs, terminal inverted repeats, etc.), and TSDs if present. Protein domains were identified using InterProScan [98] or HHpred [99] available online at EMBL-EBI or the MPI Bioinformatics Toolkit [100], and all non-canonical domains were manually inspected by visualizing multiple sequence alignment (e.g., chromatin reader and NADAR domains of *Gypsy* elements, see Supplementary Material 7: Fig. S1, S2).

A phylogeny of *Gypsy* families was produced by combining the 13 available Pol protein sequences of the manually curated elements with the dataset of 85 *Gypsy* reverse transcriptase domain sequences used for phylogenetic analysis by [42]. Sequences were aligned using mafft (L-INS-i parameters), manually trimmed to reduce the alignment to only the reverse transcriptase domain, and automatically trimmed to remove gap-rich alignment columns with trimAl v1.4.rev22 (“gappyout” model) [101]. The resulting alignment was passed to IQ-TREE v2.3.0 [102] which was run using the parameters “-m MFP -bb 1000” to perform model selection [103] and ultrafast bootstrapping [104].

The manually curated elements were incorporated to the existing TE library published in Cock et al. (2010) (available at: https://urgi.versailles.inra.fr/Data/Transposable-elements/Ectocarpus), containing 897 consensus sequences obtained running the REPET pipeline [34] on the v3 assembly of the genome. Before merging, the nomenclature of the consensus sequences was standardized (e.g., instances of “EsGypsy” were replaced with “LTR/Gypsy,” and “EsCopia” with “LTR/Copia”) and uniqueness was ensured by appending a unique identifier based on an incremental counter to each sequence header. To address misclassification, the unified library was clustered using CD-HIT-EST v4.8.1 [105] with 80% identity and length thresholds (-c 0.8 -aS 0.8 -d 0 -G 0 -g 1 -b 500 -M 8000). Following clustering, REPET consensus sequences were reclassified based on their association with manually curated elements to maintain consistency within the library.

A known issue with REPET is the misclassification of tandem repeats as nonautonomous LTRs [106]. Tandem repeats in the v5 genome were identified using Tandem Repeat Finder (TRF) v4.09.1 [107] using the parameters “2 5 7 80 10 50 2000 -d -ngs”, and resulting coordinates were subsequently intersected with the results of a preliminary RepeatMasker run (see below). For each TE family, the percentage of its cumulative length that overlapped with tandem repeats identified by TRF was calculated, and any family with more than 60% overlap was reclassified as a tandem repeat in the library.

Redundancy within the library was addressed through a two-step process. First, CD-HIT-EST v4.8.1 [105] was executed again with a 95% similarity threshold (-c 0.95 -aS 0.95 -d 0 -G 0 -g 1 -b 500 -M 8000). The clustering results were then used to identify and remove redundant sequences. To perform a final round of reclassification, remaining unclassified elements were used as queries in a blastx search with -evalue 0.001 against TE proteins from Repbase, supplemented with novel *Ectocarpus* TE proteins annotated as part of the curation process (Supplementary Material 2).

### Analysis of transposon distribution along the Ectocarpus genome

TEs were mapped on the *Ectocarpus* v5 genome [33] using RepeatMasker version open-4.0.9 (Smit et al. 2013–2015; http://www.repeatmasker.org) installed with rm-blast v2.9.0. The following parameters were set: -a -s -gff -xsmall -cutoff 230. The RepeatMasker output files were processed using the Perl script onecodetofindthemall.pl [108] with the parameters — unknown and — strict, which reconstructs fragmented repeats and full-length LTR retrotransposons, excluding tandem repeats from the analyses. After OneCodeToFindThemAll, the number of copies decreased from 233,743 detected through the annotation step with RepeatMasker to 131,728.

The output files of OneCodeToFindThemAll were concatenated in a single file, then processed through an in-house script to generate BED and TSV files providing coordinates of each TE copy. TE copies were defined as “intact” if they exhibited less than 20% divergence from their consensus sequence and spanned more than 80% of the consensus sequence length.

Tandem repeats were identified and annotated by combining the output of RepeatMasker (simple repeats and consensus sequences identified as tandem repeats, see above) with the output of TRF. When TE and tandem repeat annotations overlapped, TEs were given precedence. Computationally reproducible analysis scripts can be found at (https://github.com/edinatale/Ectocarpus-TEs.git).

The genome was divided into 100-kb non-overlapping windows and the density of TEs, tandem repeats and CDS was calculated for each window with coverageBED. To visualize the distribution of TEs, tandem repeats and CDS specifically in the male and female SDRs, 50-kb windows were used given the small size of these genomic regions. The densities in each window were plotted with Circos [109] and KaryoploteR [110]. TE density per 100-kb window was used to compare sex chromosome versus autosomes and between PAR, SDR, and autosomes using respectively the Kruskal–Wallis test followed by Dunn’s test with Bonferroni correction, and Wilcoxon rank-sum tests.

To visualize the distribution of TEs adjacent to subtelomeres, each chromosome was split to 5-kb windows where the first window begins immediately adjacent to the subtelomeric satellite repeats. For each window the median, first and third quantiles were calculated among all chromosomes. The sex chromosome was excluded from this analysis because one subtelomeric region has not been defined.

### TE location relative to genes

The *Ectocarpus* annotation was first checked against repeat coordinates to avoid including any genes carried by TEs in analyses. An in-house script was used to calculate the intersect between TE coordinates and CDS per transcript, and any transcripts exhibiting > 70% intersect with TEs were removed.

TE densities were calculated by site class by dividing the genome into 5’ and 3’ UTRs, CDS, introns, and intergenic region. Moreover, intergenic sequence was divided to gene proximal sequences (within 500 bp of a gene) or gene distal sequences. The densities calculated in Fig. 3A were obtained using BEDTools coverageBed [111]. When considering individual TE copies, a copy was defined as intergenic proximal if > 1% of its sequence fell within 500 bp of a gene. TE copies assigned as intergenic proximal, intergenic distal, and intronic were compared based on their length and their percentage of divergence from their consensus sequence, and statistical analyses were conducted using Kruskal–Wallis and Dunn’s tests.

To test the relationship between intronic TE content and gene expression, the ratio of the total genic TE content to CDS length was calculated. Mono-exonic genes were not considered in this analysis, because of their overall low expression in the *Ectocarpus* genome [27]. The evolutionary ages of *Ectocarpus* genes were estimated using GenEra [60] using default parameters.

The distance of each gene to the nearest TE was calculated using BEDTools closest [111], with intronic TEs excluded. For each gene, a maximum distance of 2 kb from the nearest TE was considered, given that the typical intergenic space is approximately 1 kb. Genes were grouped based on the distance to the nearest TE: within 500 bp, between 500 bp and 1 kb, between 1 and 2 kb, and greater than 2 kb. These groups were subsequently used to assess the relationship between the proximity of the nearest TE and the expression levels (log2 TPM) of the corresponding genes based on previous gene expression data [29, 112].

### Algae strains and growth conditions

All biological material manipulations were carried out under sterile conditions. *Ectocarpus sp7* strain Ec32 male parthenosporophytes were grown at 14 °C for 3 weeks in sterile and filtered natural seawater in 12 h/12 h dark/light cycles. When reaching sexual maturity, unilocular sporangia were manually isolated and incubated in a moist chamber for 24–48 h in high light conditions as described in [113] until spore release. Gametophytes were incubated for approximately 2 weeks until collected (immature gametophytes) and then for 4–5 weeks at 14 °C—until production of plurilocular gametangia was confirmed under microscope (fertile stage). Gametes were span at 10,000* g* for 5 min; supernatant was removed carefully without disturbing the gamete pellet which was flash frozen in Liquid Nitrogen and stored at − 80 °C until RNA extraction was performed. Approximately, 1500 gametophytes (150 Petri dishes with 10 gametophytes each) were used for gamete release per biological replicate. Immature and fertile gametophyte tissue was collected from 5 Petri dish with 10 gametophytes each at the same time of development. Immature gametophytes were grown in the same conditions but harvested 3 weeks before the gametangia development and lack of plurilocular sporangia was confirmed under the light microscope. Three independent biological replicates were collected for each sample, except for the gametes in which 2 biological replicates were collected.

### sRNA library preparation and short-read sequencing

sRNA libraries were prepared starting with total RNA isolated from 50–100 mg of tissue, except for the “gamete” sample in which it was not possible to estimate weight. Instead, all gametes harvested from one gamete release from approximately 1500 gametophytes were used. Total RNA was isolated as previously described [30]. Briefly, gametophytes were grounded to a fine powder in liquid nitrogen in 1.5 mL Eppendorf tubes using a micropestle. Gametes were directly resuspended in the extraction buffer by up and down pipetting as well as vertexing. The algae powder was further homogenized in 500 μl of cetlytrimethylammonium bromide (CTAB)-based extraction buffer [100 mM Tris–HCl pH 8, 1.4 M NaCl, 20 mM EDTA pH 8, 2% Plant RNA Isolation Aid (PVP, Invitrogen AM9690), 2% CTAB and 1% β-mercaptoethanol by vortexing and incubating at 65 °C until all samples were processed (5–20 min). RNA was extracted by mixing the homogenate with 1:1 volume of chloroform/isoamylalcohol (24:1). Supernatant was collected after centrifugation at 10,000* g* for 15 min at 4 °C and the chloroform/isoamylalcohol extraction step was repeated. RNA was precipitated with 1:1 (v/v) isopropanol at − 20 °C overnight. Samples were centrifuged at 20,000* g* for 30 min at 4 °C and pellets washed twice with 1 mL of 70% ethanol and resuspended in nuclease free water. DNase treatment was performed using a TURBO DNase Kit for 20 min, according to the manufacturer’s instructions (Thermo Fisher Scientific, AM1907). Total RNA was purified with the RNA clean and concentrator kit (Zymo Research, R1013) following the manufacturer’s instructions with the following modifications: columns were washed twice with 400 μl of RNA Prep buffer and four times with 700 μl of RNA Wash Buffer. RNA was eluted in 12 µL of nuclease-free water. sRNA libraries were prepared as described in [114] starting from 1 µg of total RNA. In brief, NEBNext multiplex small RNA library prep kit (New England Biolabs) was used according to the manufacturer’s instructions. Libraries were indexed during the PCR step with 15 cycles and size-selected using BluePippin 3% agarose cassettes (Sage Science). Equimolecular pooled libraries were sequenced with Illumina NextSeq2000 on a 1 × 50 bp single-end reads. The sRNA sequencing data can be accessed in the SRA Knowledge Base with the accession number PRJNA1197354.

### sRNA-sequencing analysis

The small RNA reads generated (Supplementary Material 8: Table S19) in triplicate for the vegetative (S1, S2, S3) and fertile (S4, S5, S6) gametophytes, and in duplicate for gamete samples (S7, S8), were processed using snakemake_sRNAseq (https://github.com/seb-mueller/snakemake_sRNAseq), an automated Snakemake [115] pipeline for small RNA-sequencing data analysis. Read quality was assessed with FastQC (v0.11.7, http://www.bioinformatics.babraham.ac.uk/projects/fastqc/). Adaptor sequences were removed from raw reads using cutadapt (v4.2; [116] with quality trimming (-q 20) and length filtering flags.

Two independent mapping analyses were performed. In the first step, reads < 18 or > 40 bp were discarded (length filtering -m 18 -M 40), and the remaining sequences were aligned to the Ectocarpus sp. 7 reference genome [33] and the TE consensus sequences. In the second step, only reads between 20 and 24 bp (length filtering -m 20 -M 24) were selected and mapped to the same reference genome. Mapping was performed with bowtie version 1 [117], using the parameters “multi” or “unique”, -v 0, -m 1, optimized for high-accuracy alignment of short reads [118]. Both uniquely and multi-mapped reads were retained for downstream analysis. The configuration files used for this analysis are available at 10.17617/3.4SCUJN.

Small RNA abundance was quantified as counts per million (CPM) over individual TE copies using the featureCounts function from the R package Subread [119], with the options countMultiMappingReads = TRUE, allowMultiOverlap = TRUE and fracOverlap = 1. Differential accumulation of sRNAs over TEs was assessed with DESeq2 [120] using raw count data. Raw read counts were filtered to retain only TE loci with a minimum of 10 reads across all samples, and shrinkage was applied using the apeglm method. Comparisons were conducted separately for the following groups: vegetative gametophyte vs. fertile gametophyte, vegetative gametophyte vs. gametes, and fertile gametophyte vs. gametes.

### Chip-seq mapping and analysis

FastQ files for the ChIP-seq data (H3K79me2) from *Ectocarpus* male gametophyte were downloaded from the SRA database [29]. Data were mapped to the V5 reference genome and the TE consensus annotated genes using the nf-core/chipseq v2.0.0 [121] with BWA as the aligner, allowing multi-mapping (“keep_multi_map”: true). To normalize the H3K79me2 ChIP-seq signal against the H3 control, bamCompare from the DeepTools suite [122] was used with a bin size of 10 bp (–bs 10) to calculate log2(IP/H3) ratios (–operation log2). The resulting normalized signal files (bigWig format) were used to compute signal intensities over annotated TEs using computeMatrix in scale-regions mode. Parameters included -bs 10, -b 10,000, -a 10,000, –regionBodyLength 1000. The flanking regions of 10 kb were used to ensure inclusion of non-TE sequence, thereby reducing noise in the analysis, as TEs are often located in close proximity to each other. Profiles showing the distribution of normalized ChIP-seq signal over TEs were generated using plotProfile, with the –perGroup option to create separate profiles for each TE superfamily.

Broad peaks of H3K79me2 enrichment were identified using MACS2 [123] with the –broad option, and using the corresponding H3 ChIP-seq data as the control. Peak files were intersected with TE annotations using BEDTools [111] to calculate the proportion of each TE copy overlapping with H3K79me2-enriched regions.

### TE expression

RNA-seq data from the wildtype male *Ectocarpus*, encompassing the vegetative gametophyte, fertile gametophyte, and gamete stages, was obtained from [124] and mapped to the *Ectocarpus* V5 reference genome using the nf-core/rnaseq v3.12.0 [121] with default parameters. TE expression levels were quantified using TEspeX [125], and transcript abundance for each TE family was calculated in transcripts per million (TPM), normalized by the length of the consensus sequence representing each family.

## Supplementary Information

Supplementary Material 1.

Supplementary Material 2.

Supplementary Material 3.

Supplementary Material 4.

Supplementary Material 5.

Supplementary Material 6.

Supplementary Material 7: Figures S1-S7.

Supplementary Material 8: Tables S1-S19.

## Data Availability

The transposable element consensus sequences are available on Figshare (10.6084/m9.figshare.29832329). The sRNA sequencing data can be accessed in the SRA Knowledge Base (https://www.ncbi.nlm.nih.gov/sra) with the accession number PRJNA1197354, which also corresponds to the associated BioProject https://www.ncbi.nlm.nih.gov/bioproject/1197354. Supplementary Material and scripts are available in GitHub at https://github.com/edinatale/Ectocarpus-TEs.
