# Supplementary material for: Characterization of the transposable element landscape shaping the Ectocarpus genome
Source: Genome Biol. 2025 Sep 29;26:320. doi: 10.1186/s13059-025-03742-z (PMC12477816; doi:10.1186/s13059-025-03742-z)
Supplement: Supplementary file 7 — Supplementary Material 7: Figures S1-S7. [file 13059_2025_3742_MOESM7_ESM.pdf]

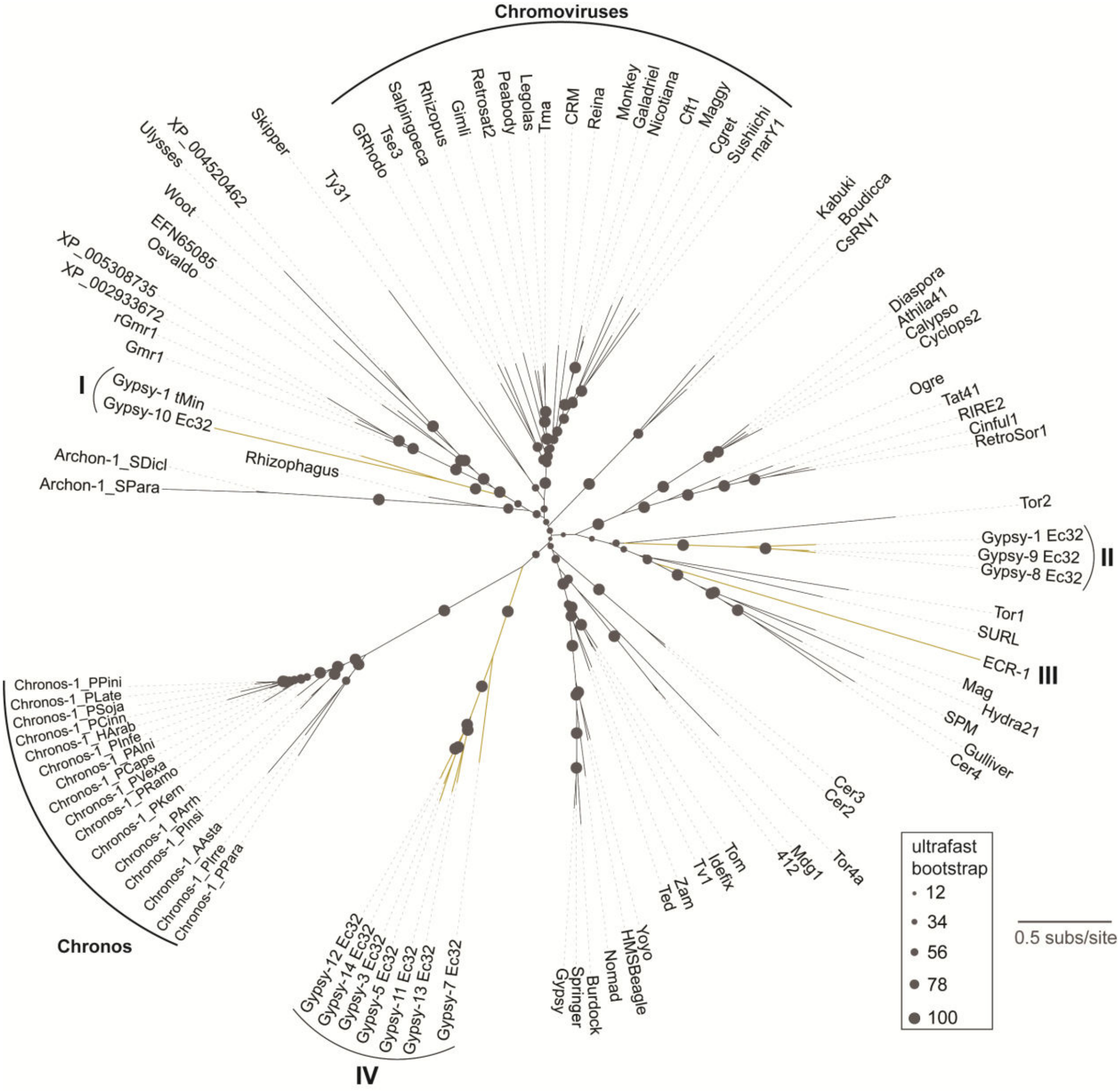


```
I    Gypsy-10_Ec32 1869 VGTKVRQSFTDG--GGQ-VSEqtgmvYDFREPYWKVRYPDGDWEELSRREVN 1917
     Gypsy-1_tMin  2089 RGTKVAREHRDS--LKQrVKYvwgtvVGYLAPYWRVRYEDGEWEDFTKRQLQ 2138
     cd20401          1 VGRRVRKKFDGEwfDGT-VVS-----YDKKTGLYHVEYEDGDAEELTEDELR 45

II   Gypsy-1_Ec32  1189 YVVEGIDKFKRGAGKPSlhnfqYYFLLKDR-PASLGCWKHFKEVPQCHEMIRSYR 1242
     Gypsy-8_Ec32  1327 YVVLKVVGHRSGQGRRGphnyrYRLRLKGY-GPESDLMYRADEVPQCHEMISAYR 1380
     Gypsy-9_Ec32  1343 YVVDKVVSHKSGKGPKTpynykYRLRLRGY-GPESDLEYRADEIPQCQEMISAYR 1396
     cd18961          1 YEVEKILSHRIVNGKPL-----YLVMWVGYpGPVENSEMWEEDLKNCGELLKAYK 50

III  ECR-1         1626 EFEMAAIVDMSVAEDGPGFDVEVEWVGFDKEENTWEELSKVWDAAPQ 1672
     smart00298       1 EYEVEKILDHRWKKKG-ELEYLVKWKGYSYSEDTWEPEENLLNCSKK 46

IV   Gypsy-7_Ec32  2031 -KISRHRICRGISGKGAVQYYTHWTGLAK--CTWEHEVELEQYGDVVLKY 2077
     Gypsy-3_Ec32  2331 EKITGHQSVRGRGGVIAVMYETHWKGLLR--PSWEREMDLQHSRKQILLY 2378
     Gypsy-5_Ec32  2228 EKITGHQTVRGRGGVLAALYQTHWKGLLR--PSWEREIDLQHSRHHILRY 2275
     Gypsy-11_Ec32 1960 DYISGHQLVRGRSGVLAVLYQTHWLGLTT--PSWERESDLDQFRRHILLY 2007
     Gypsy-12_Ec32 1991 DRISGHQSVRGRGGTIAVLYQTHWKGLLR--PSWEREMDLQHSKPHILRY 2038
     Gypsy-14_Ec32 2240 EQITGHQSVRGRGGVIAVLYQTHWKGLLR--PSWEREMDLQHSRSQILRY 2287
     smart00298       1 EYEVEKILDHRWKKKGELEYLVKWKGYSYseDTWEPEENLLNCSKKLDNY 50
```

Fig. S1. A) Maximum likelihood phylogeny of *Gypsy* LTR retrotransposons based on the reverse transcriptase domain of the pol protein, using the LG+R7 mode. Curated proteins from *Ectocarpus* and *T. minus* were combined with the dataset from Ustyantsev et al. 2017. The chromodomain-encoding Chromovirus and Chronos clades are

highlighted (note that the support value of the node that unites Chronos and *Ectocarpus* lineage IV elements is low). B) Alignment of *Ectocarpus* and *T. minus* putative chromatin reader domains to CDD and Pfam domains. cd20401 = Tudor domain superfamily; cd18961 = CEC-4_like chromodomain; smart00298 = chromodomain.

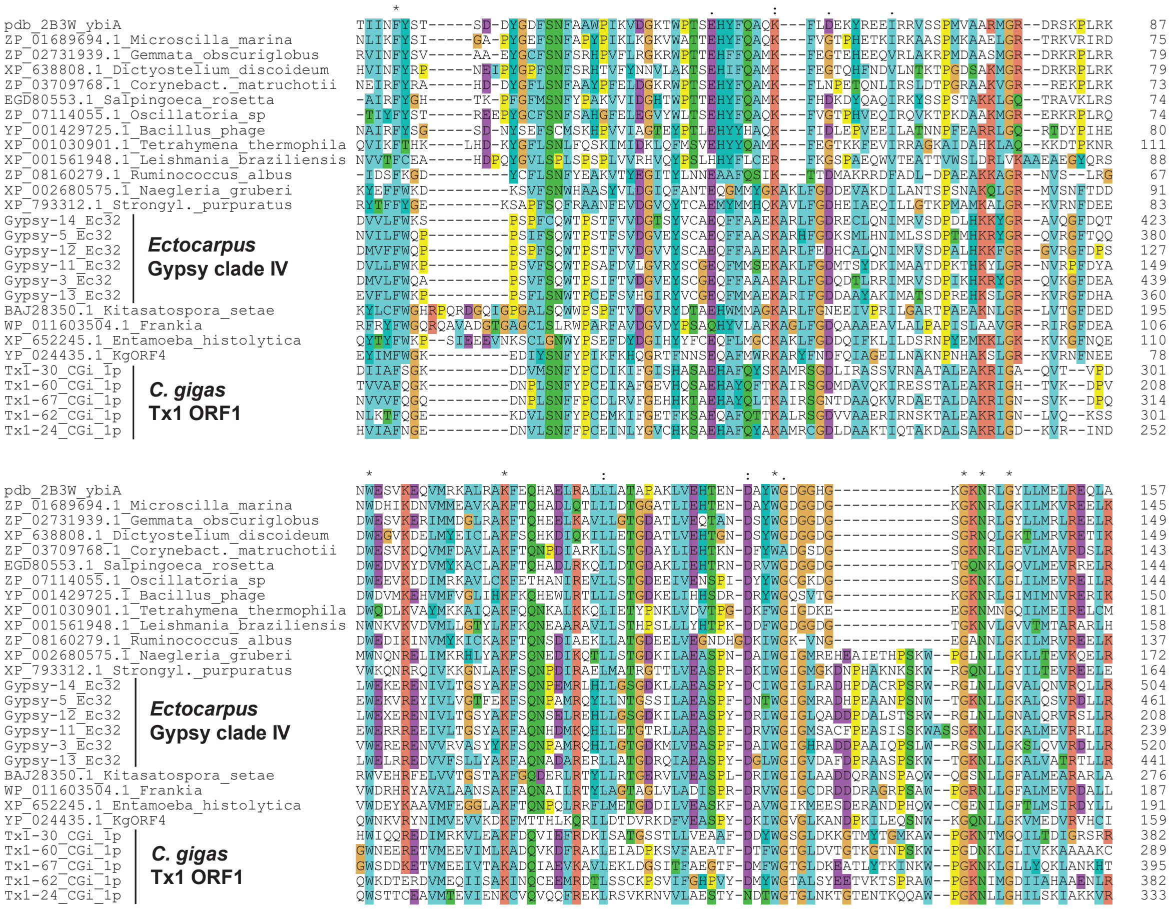

Fig. S2. Alignment of YbiA-like NADAR domains, including N-terminal domains from pol proteins of lineage IV *Ectocarpus Gypsy* LTR retrotransposons and domains from ORF1 of *Tx1* LINEs from *C. gigas*. Protein sequences were taken from de Souza & Aravind (2012).

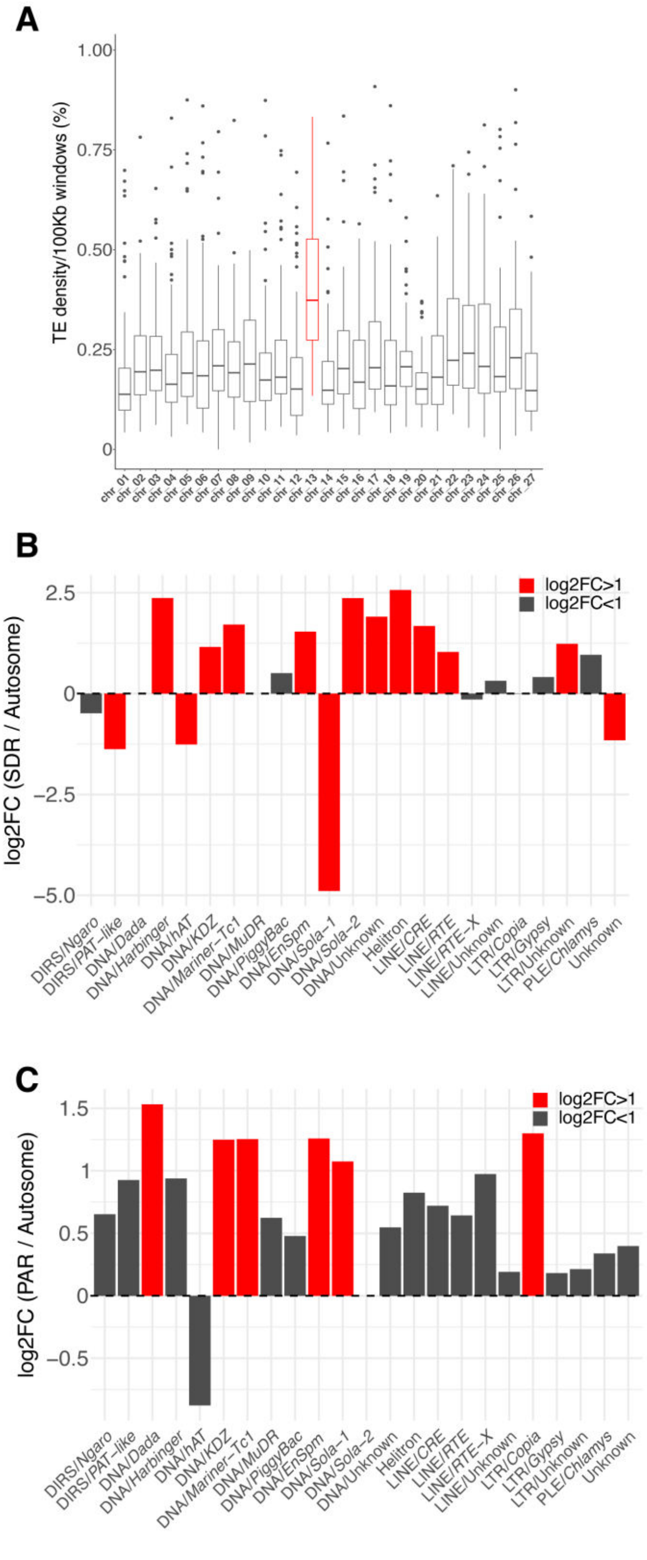

Fig. S3. (A) TE coverage on each chromosome; the autosomes are showed in gray, while the sex chromosome is highlighted in red. (B) Log_2 fold change of TE superfamily densities on the sex-determining region (SDR) versus autosomes. (C) Log_2 fold change of TE superfamily densities on the pseudoautosomal region (PAR) versus autosomes.

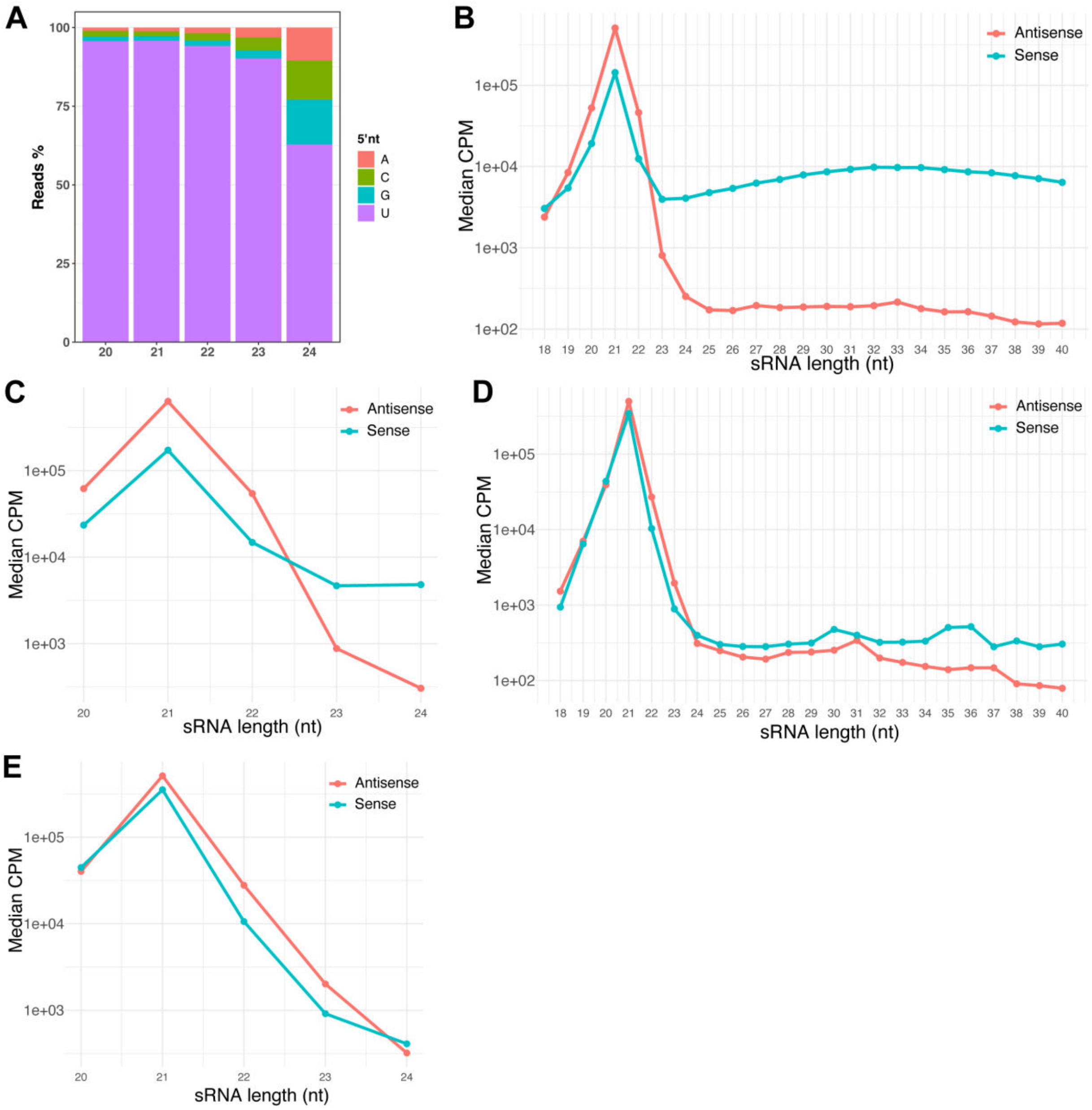

Fig. S4 (A) sRNA nucleotide composition at the 5' end for TEs in *Ectocarpus*. (B) Size distribution (18-40 range) and orientation (sense/antisense) of sRNA mapping on CDS in the *Ectocarpus* genome. (C) Size distribution (20-24 range) and orientation (sense/antisense) of sRNA mapping on CDS in the *Ectocarpus* genome. (D) Size distribution (18-40 range) and orientation (sense/antisense) of sRNA mapping on TE copies in the *Ectocarpus* genome. (E) Size distribution (20-24 range) and orientation (sense/antisense) of sRNA mapping on TE copies in the *Ectocarpus* genome.

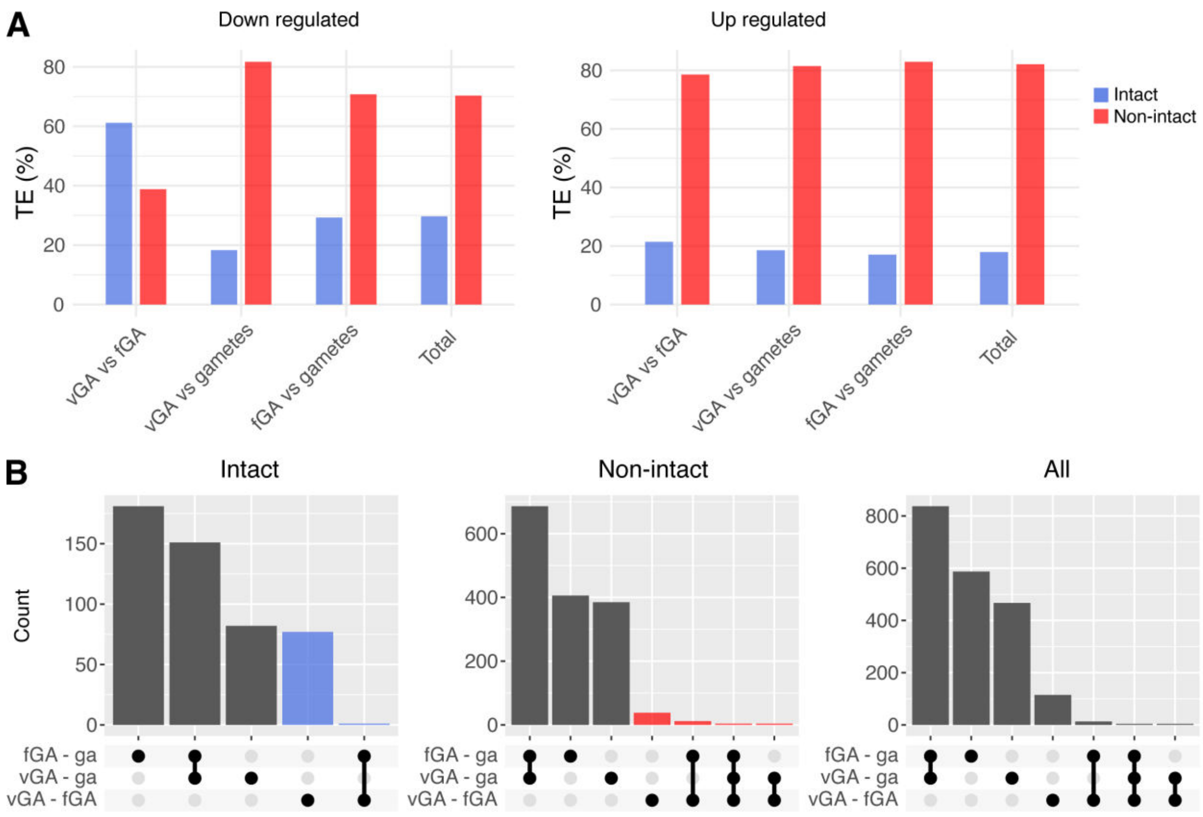

Fig. S5 (A) Bar plot displaying the proportion of intact (blue) and non-intact (red) TEs that are either down-regulated (left panel) or up-regulated (right panel) across different life stages of Ectocarpus (B) Upset plot showing the intersection between down-regulated TEs across the three developmental transitions analyzed.

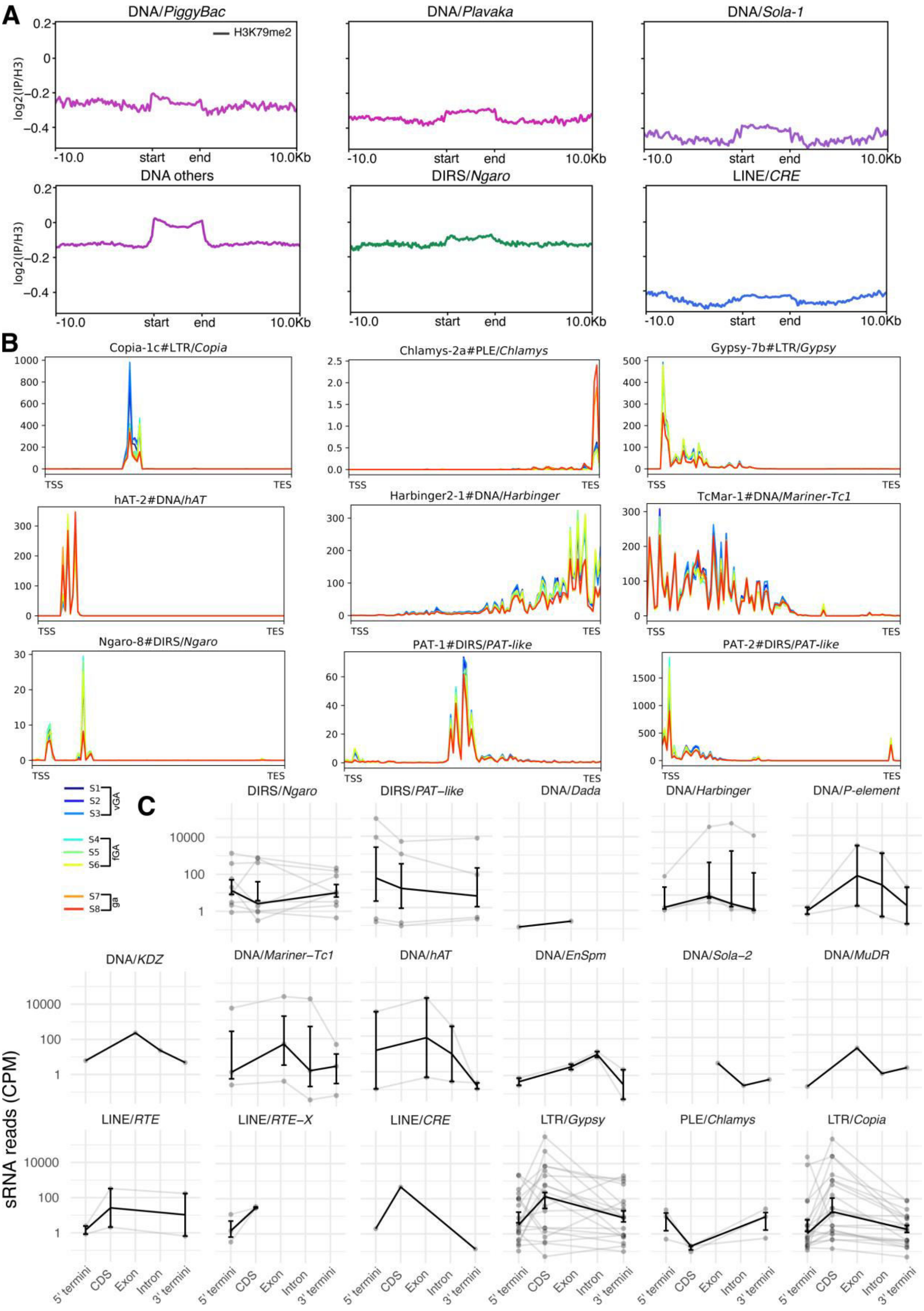

Fig. S6 (A) H3K79me2 (normalized on H3) accumulation profiles on TE superfamilies. Start and end indicate the TE body, with 10 kb flanking regions**.** (B) sRNA reads distribution over TE body. Each line represents a biological replicate. (C) sRNA reads (CPM) over TE features. Each gray dot represents the mean CPM value, across 8 biological replicates, for each TE family

consensus sequence. Gray lines connect dots belonging to the same TE family. The black line represents the median CPM value for each TE feature, with SD, within the TE superfamily represented.

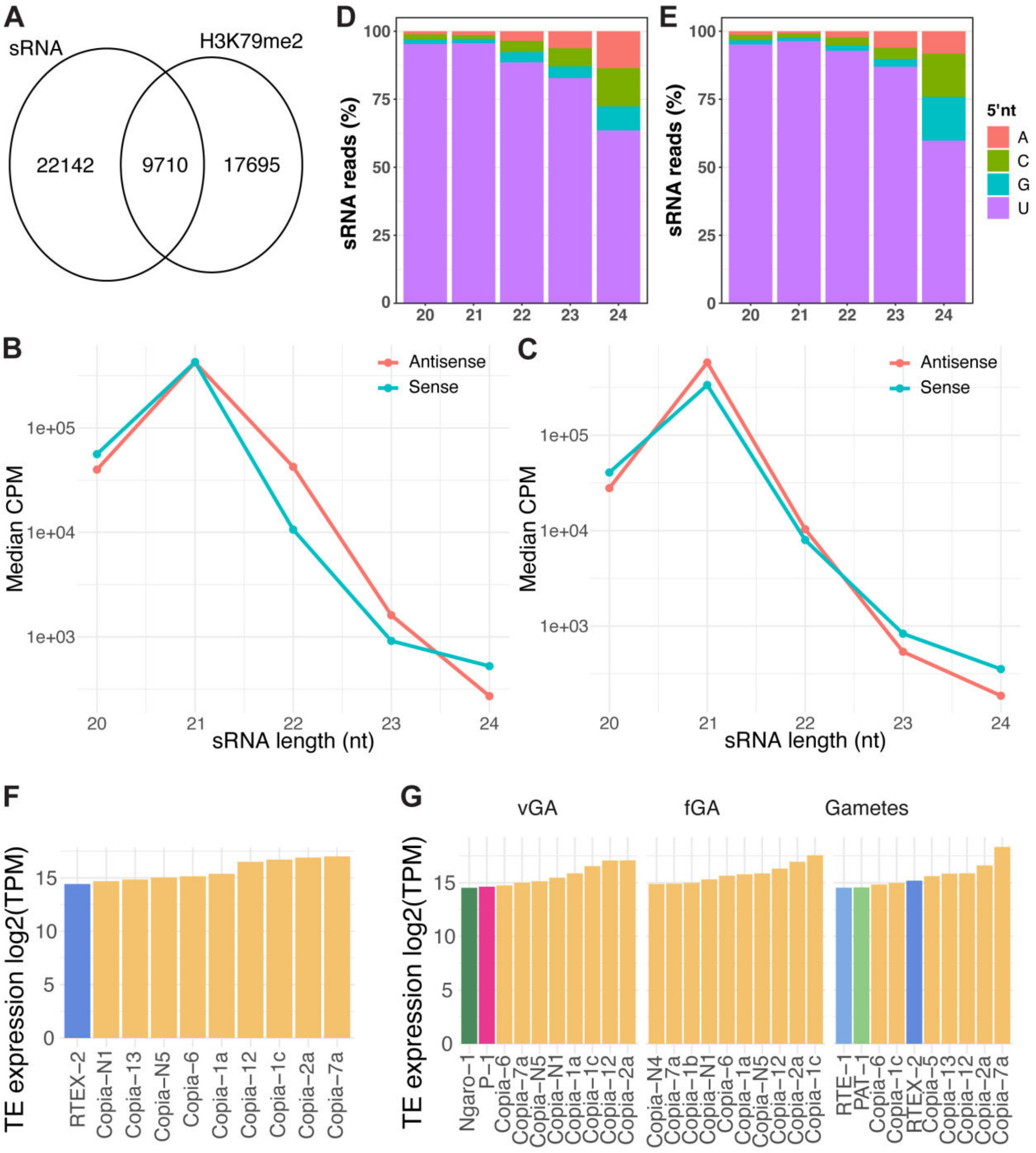

Fig. S7 (A) Venn diagram showing the intersect between TE copies associated with sRNA and H3K79me2. (B) Size distribution and orientation (sense/antisense) of sRNA reads (mean CPM across 8 biological samples) mapping over intact TEs overlapping with H3K79me2 peaks. (C) Size distribution and orientation (sense/antisense) of sRNA reads (mean CPM across 8 biological samples) mapping over intact TEs not overlapping with H3K79me2 peaks. (D) sRNA nucleotide composition at the 5' end for intact TEs that overlap with H3K79me2 peaks. (E) sRNA nucleotide composition at the 5' end for intact TEs that not overlap with H3K79me2 peaks. (F) TE gene expression (TPM) of the ten most expressed families overall. (G) TE gene expression (TPM) of the ten most expressed TE families in the three life stages taken into consideration (vegetative gametophyte, fertile gametophyte, gametes).
